# Early Onset of Nucleate Boiling on Gas-covered Biphilic Surfaces

**DOI:** 10.1038/s41598-017-02163-8

**Published:** 2017-05-17

**Authors:** Biao Shen, Masayuki Yamada, Sumitomo Hidaka, Jiewei Liu, Junichiro Shiomi, Gustav Amberg, Minh Do-Quang, Masamichi Kohno, Koji Takahashi, Yasuyuki Takata

**Affiliations:** 1 International Institute for Carbon-Neutral Energy Research (WPI-I2CNER), Kyushu University, 744 Motooka, Nishi-ku, Fukuoka 819-0395 Japan; 2 Department of Mechanical Engineering, Kyushu University, 744 Motooka, Nishi-ku, Fukuoka 819-0395 Japan; 3 Department of Mechanics, The Royal Institute of Technology, S-100 44 Stockholm, Sweden; 4 Department of Mechanical Engineering, The University of Tokyo, 7-3-1 Hongo, Bunkyo-ku, Tokyo 113-8656 Japan; 5 Department of Aeronautics and Astronautics, Kyushu University, 744 Motooka, Nishi-ku, Fukuoka 819-0395 Japan

## Abstract

For phase-change cooling schemes for electronics, quick activation of nucleate boiling helps safeguard the electronics components from thermal shocks associated with undesired surface superheating at boiling incipience, which is of great importance to the long-term system stability and reliability. Previous experimental studies show that bubble nucleation can occur surprisingly early on mixed-wettability surfaces. In this paper, we report unambiguous evidence that such unusual bubble generation at extremely low temperatures—even below the boiling point—is induced by a significant presence of incondensable gas retained by the hydrophobic surface, which exhibits exceptional stability even surviving extensive boiling deaeration. By means of high-speed imaging, it is revealed that the consequently gassy boiling leads to unique bubble behaviour that stands in sharp contrast with that of pure vapour bubbles. Such findings agree qualitatively well with numerical simulations based on a diffuse-interface method. Moreover, the simulations further demonstrate strong thermocapillary flows accompanying growing bubbles with considerable gas contents, which is associated with heat transfer enhancement on the biphilic surface in the low-superheat region.

## Introduction

Pool boiling, one of the most common and ubiquitous phase-change phenomena, finds extensive application in a wide range of energy solutions from air-conditioning and refrigeration to nuclear and fusion reactor cooling^1–4^. Thanks to large latent heat of vaporization and, arguably more importantly, strong agitation effect (induced by sizable change in specific volume during phase change), heat can be dissipated much more efficiently by way of boiling than through either single-phase conduction or convection alone^2, 5^. Evaporation of liquid leads to repeated cycles of bubble nucleation, growth, coalescence, and departure from the heat transfer surface, which enables transport of significant amounts of heat at modest surface superheats (defined as excess temperature increases relative to the saturation temperature)^4^. Notwithstanding continued interest, boiling heat transfer (BHT) as yet defies a complete physical description^5–8^. For boiling is inherently a multi-physics phenomenon, dependent not only on thermohydrodyamics of the liquid phase but also on numerous complex interfacial sub-processes, the fundamental mechanisms of BHT are still not fully understood^7^. Obtained from a rapidly growing wealth of experimental and numerical studies^9, 10^, one emerging consensus concludes that transient heat conduction, microconvection, and microlayer evaporation all contribute to the overall boiling performance—albeit to varying extents—of which a unified mechanistic model remains elusive^11^. Moreover, vital correlations can be made between BHT and a large collection of surface characteristics, including roughness^8, 12, 13^, texture^14, 15^, topography^16^, porosity^3, 17^, and wettability^18–21^ in particular, adding to the intractableness of the problem.

The range of effectiveness of boiling as a reliable heat transfer scheme is determined by the critical heat flux (CHF) and the onset of the nucleate boiling (ONB). The former denotes the upper limit of nucleate boiling, whereupon a potentially devastating transition to the far less desirable regime of film boiling could ensue. Such a boiling crisis entails sudden and dramatic deterioration of heat transfer as a result of unimpeded bubble merging and formation of an insulating vapour layer. The physical interpretations concerning CHF have been developed through the prisms of the hydrodynamic theory^22, 23^ (i.e., Taylor-Helmholtz instabilities), the evaporation momentum force model^24^, and the thermal-hydraulic model^2^ (describing the dynamic competition between the dry-out and rewetting timescales). The latter constraint, on the other hand, involves boiling incipience. For electronics cooling applications, delayed ONB could cause thermal shocks, which are particularly detrimental to the long-term system stability^4^. According to the prevailing heterogeneous nucleation model^9^, bubbles tend to grow from pre-existing vapour embryos trapped in the defects of the boiling surface. Hence, both sufficient surface roughness and liquid superheating (relative to the saturation temperature) have long been considered necessary conditions for bubbles to overcome the interfacial energy barrier and emerge from the mouth of the crevice.

However, increasing evidence mounts from recent experiments challenging the pre-eminence of such explanations that vapour-trapping cavities  act as preferred nucleation sites. Bourdon *et al*.^25^ used ultra-smooth featureless glass surfaces to essentially suppress the influence of surface topography on ONB. Specifically, by chemically grafting SAM (self-assembled layers) onto the surface, they showed a dramatic decline of the incipient boiling superheat with decreasing wettability. Hypotheses such as the presence of nanobubbles^26^ and accelerated molecular mobility (leading to greater chances of initiating spontaneous phase change)^27^ on hydrophobic surfaces were advanced as alternative nucleation mechanisms that can possibly account for the apparent deviation from the classical nucleation theory. Recently, the second requirement of liquid superheating has too been called into question by a series of unexpected observations of unusual nucleation events associated with surface hydrophobicity. Namely, the initiation of nucleate boiling was found to occur at extremely low surface temperatures^28, 29^ and in some extreme cases, even below the minimum liquid-vapour phase-change temperature predicted by thermodynamic arguments^30, 31^. The surprising results were tentatively attributed without a concrete proof to a considerable amount of dissolved air that somehow precipitated out of the liquid and got mixed with vapour to induce heterogeneous bubble generation. Still, lingering doubts remain since a rigorous degassing procedure was purposely carried out prior to the experiment to ensure very low levels of incondensables in the liquid^32^.

In this paper, we present new unequivocal evidence for the critical role of dissolved gas in the early onset of boiling on a hydrophobic surface. It is found that for a heterogeneous biphilic surface (that is, with a spatially-alternating wettability pattern), positive boiling incipient superheats can be recovered when vacuum degasification, instead of simple boiling deaeration, is adopted. The assumed differential presences of dissolved gas are further confirmed through a comparison with diffuse-interface predictions of contrasting bubble dynamics of a pure vapour bubble and a vapour-gas bubble mixture. In addition, the simulations show that the high gas contents in the bubble give rise to a strong thermocapillary effect, which is deemed partially responsible for moderate heat transfer enhancement in the low-superheat region.

## Results

### Boiling deaeration versus vacuum degasification

**Figure 1:**
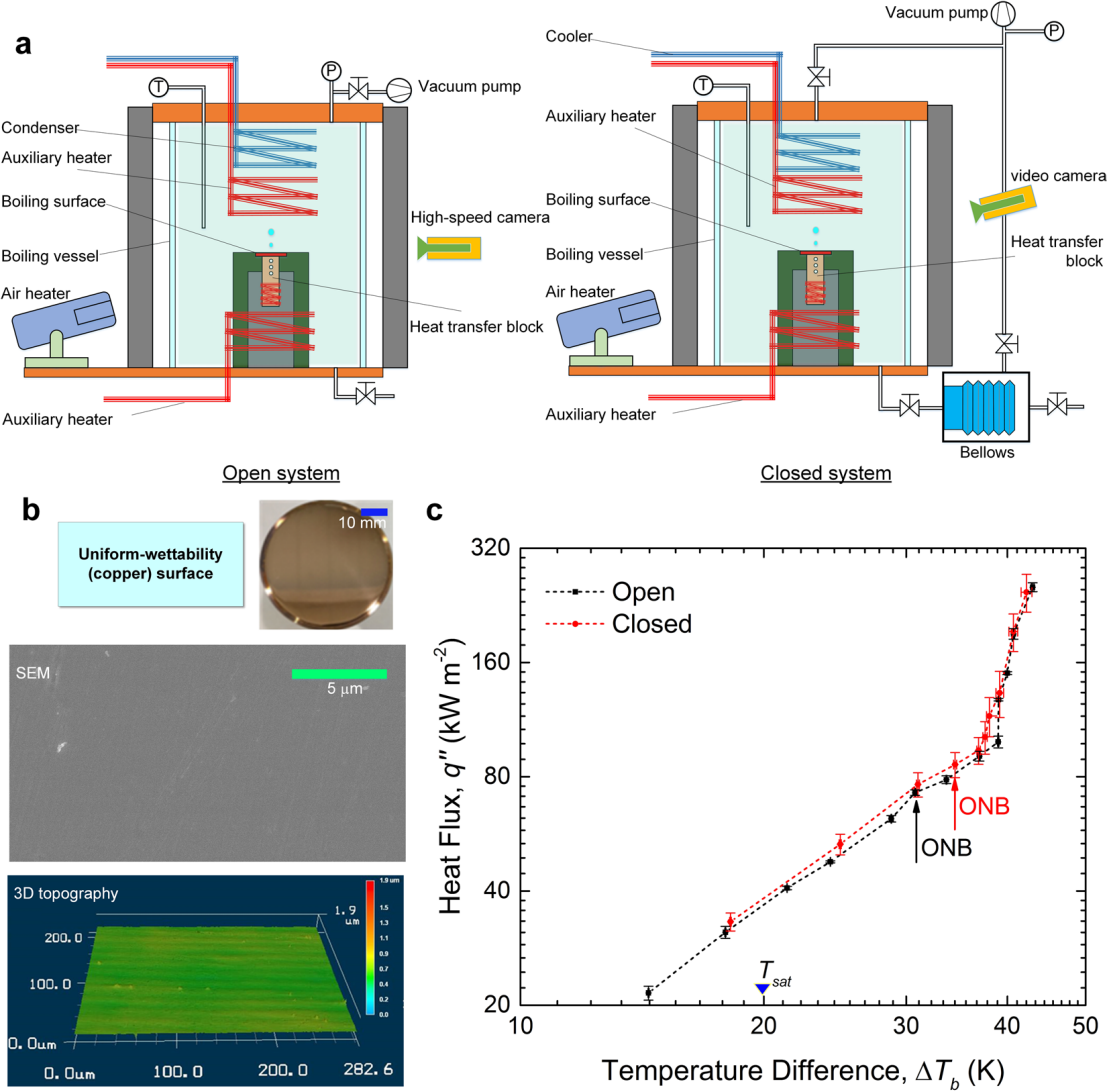
Pool boiling experiments using open and closed setups. (**a**) Boiling experimental apparatuses of the open and closed systems. Instead of boiling deaeration, more thorough vacuum degasification was employed in the closed system. The content of dissolved air in subcooled water (*T*_*bulk*_ = 80 °C) decreased from *c*_*g*_ ≈ 5.53 ppm in the open vessel to *c*_*g*_ < 0.7 ppm in the closed system that can be hermetically sealed off from the atmosphere, thanks to a pressure-stabilizing rubber bellows. Heat was incrementally applied to the heat transfer block to generate boiling on its upward-facing surface of 30 mm in diameter. The surface temperature and heat flux was calculated by a heat conduction model based on steady-state measurements of the three thermocouples embedded along the length of the heat transfer block. (**b**) A homogeneous boiling surface with uniform wettability. The plain copper surface with a contact angle ≈80°, polished to a mirror finish, was largely free of surface defects as shown by the scanning electron microscope (SEM) image. The surface morphology measurement by a 3D laser profilometer shows an average roughness of 0.03 µm. **(c)** Plot of the boiling curves obtained for the homogeneous surface. The boiling incipience superheat (Δ*T*_*sat*_ = *T*_*w*_ *-T*_*sat*_) rose slightly from Δ*T*_*sat*_ = 12.8 K in the open system to Δ*T*_*sat*_ = 15.2 K in the closed system. Error bars represent standard deviations of the least-square fittings. Despite the somewhat lower ONB in the open system, BHT varied little following the dissimilar degassing schemes in the present study.

To characterize the effect of dissolved gas on bubble generation, two boiling experimental apparatuses with different degassing procedures have been built (Fig. 1a). Both setups comprise of a cylindrical boiling vessel and employ two auxiliary coil heaters and a condenser (cooler) to control the bulk temperature, *T*_*b*_. The key difference between them lies in their capability for active pressure control. Connected to a rubber bellows encased in a vacuum chamber, one boiling vessel (referred hereafter to as the *closed* system) can be hermetically sealed off from the surroundings while maintaining pressure at a prescribed (for instance, atmospheric) level. The second boiling setup (denoted hereafter as the *open* system), for lacking this feature, needs to stay constantly open to the atmosphere lest pressure gradually builds up once boiling starts. Deionized water was the working fluid. No chemical analysis has been performed to determine the exact composition of the bulk liquid. Instead, we estimate the amount of air dissolved in water using Henry’s law: *c*_*g*_ = *HP*_*g*_ = *H*[*P*_*t*_ − *P*_*sat*_(*T*_*sat*_)], where *c*_*g*_ is the concentration of gas in the aqueous solution and *H* is the solubility constant. The gas partial pressure in the gaseous phase, *P*_*g*_, is considered equal to the difference between the total (system) pressure, *P*_*t*_, and the saturated vapour pressure, *P*_*sat*_, at the corresponding temperature, *T*_*sat*_. Assuming air to be a binary mixture of 21% oxygen (${H}_{{O}_{2}}$|_*25*__*°C*_ = 1.3 × 10^−3^ mol L^−1^ atm^−1^) and 79% nitrogen (${H}_{{N}_{2}}$|_*25*__°*C*_ = 6.1 × 10^−4^ mol L^−1^ atm^−1^), we calculate an initial concentration of air in the water ≈7.31 × 10^−4^ mol L^−1^ (or 21.2 ppm). For the open system, conventional water deaeration was deployed by continuously boiling the water for over 30 minutes. Subsequently, the bulk water was allowed to cool down to 80 °C (namely, with a subcooling level of 20 K), at which point air inevitably seeped back to the water through the exposed liquid-gas interface. With ${H}_{{O}_{2}}$|_*80 °C*_ = 5.3 × 10^−4^ mol L^−1^ atm^−1^ and ${H}_{{N}_{2}}$|_*80*__°*C*_ = 3.1 × 10^−4^ mol L^−1^ atm^−1^, the equilibrium concentration of dissolved air could ultimately rise to around 1.90 × 10^−4^ mol L^−1^ (or 5.53 ppm)—at a particularly slow rate notwithstanding (Supplementary Note). It is thus safe to assume a feeble presence of air in the bulk water over the course of the experiment. On the other hand, more thorough gas removal was performed in the closed system by means of continuous vacuum degasification, which could, with a 30-fold pressure reduction, result in an even more diminutive (<0.7 ppm) amount of remaining air in the water. Additionally, on account of the airtightness of the boiling vessel, the gas level was expected to stay low during the following vessel re-pressurization—by opening the bellows chamber to the atmosphere—and preheating of the water up to the same subcooling degree of 20 K. More detailed descriptions of the experimental facilities and degassing procedures can be found in the Methods section.

Comparative pool boiling experiments with identical parameters were carried out using these two systems. Once the degassing procedure was finished, heat was applied to a copper heat transfer block placed inside the glass vessel in an incremental manner, which induced boiling on its flat upward-facing surface. The steady-state temperature, *T*_*w*_, and heat flux, *q*”, of the heat transfer surface were evaluated based on the temperature readings of three embedded thermocouples using Fourier’s law of heat conduction. Additionally, high-speed photography was employed to record the evolution of bubble dynamics over various stages of nucleate boiling. See the Methods section and Supplementary Note for more details regarding the test procedures and the uncertainty analysis. As a reference, we first performed the subcooled boiling experiments on a plain copper surface (Fig. 1b). Polished to a mirror finish, the sample surface has an average roughness of *Ra*_*copper*_ < 0.03 μm and modest affinity for water (with contact angle, *θ*_*e*_ ≈ 80°). In Fig. 1c, we plot the boiling curves (namely, the measured “*q*” vs. the temperature difference, Δ*T*_*b*_, between *T*_*w*_ and the bulk water temperature, *T*_*bulk*_) for the open and closed systems, respectively. The results show a somewhat delayed formation of bubbles in the closed system. As a demonstration of the effectiveness of both boiling deaeration and vacuum degasification in eliminating residual gas, the boiling curves for these two cases nearly overlap with each other, which agrees with past findings of only limited impact on heat transfer by dissolved air below a certain critical threshold^29, 33, 34^.

### Enhanced heat transfer due to dissolved gas

**Figure 2:**
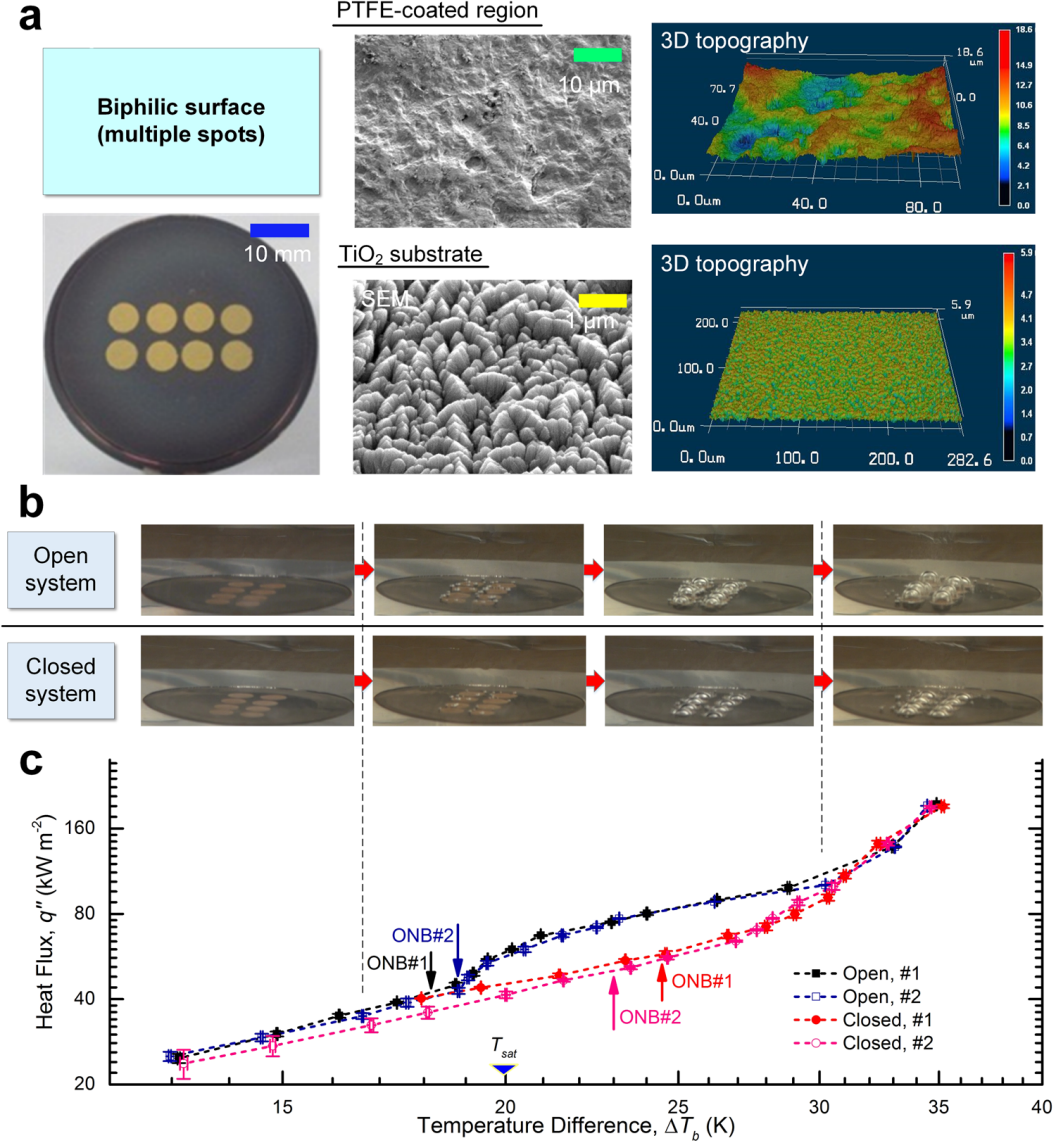
Effect of dissolved gas on boiling heat transfer on a biphilic surface. (**a**) A heterogeneous surface with mixed wettability. The superhydrophilic TiO_2_ substrate (after 12 hours of UV irradiation, *θ*_*e*_ ≈ 0°) was coated with an array of hydrophobic (*θ*_*e*_ > 120°) PTFE circular spots of 6 mm in diameter and 7 mm in pitch. The SEM and 3D laser profilometer images reveal contrasting surface topographies between the TiO_2_ and PTFE subregions, which had an average roughness of 0.3 µm and 3 µm, respectively. (**b**) Evolution of the boiling characteristics. Following the initial quiescent stage of natural convection, small bubbles first appeared around the interface between the hydrophilic and hydrophobic subregions, which then coalesced and formed a single bubble completely encompassing each hydrophobic island. Although the expansion of the bubble base was limited to the edge of the hydrophobic coatings, the mostly stationary bubbles grew notably larger in the open system due to the presence of dissolved gas. At high superheats, more bubbles started to nucleate on the TiO_2_ surface as well, which quickly departed from the surface. **(c)** Plot of the boiling curves. The open and closed cases differed in the incipience of boiling: Δ*T*_*sat*_ = 4.6 K (run #1) and 2.9 K (run #2) in the closed system, compared with Δ*T*_*sat*_ = −1.6 K (run #1) and −1.4 K (run #2) in the open system. Error bars represent standard deviations of the least-square fittings. An increasing divide seems to emerge between the boiling curves before re-converging in the regime of fully-developed nucleate boiling, which is in large part attributable to particularly strong dissolved gas-induced thermocapillary convection under the open condition.

Next, the pool boiling experiment was repeated but with a biphilic surface instead, which consists of a 4 × 2 array (with a 7-mm pitch) of hydrophobic PTFE (Polytetrafluoroethylene, *θ*_*e*_ > 120°) 6-mm circular spots deposited on a superhydrophilic TiO_2_ substrate (Fig. 2a). It should be noted that the PTFE coating manages to create as clear a contrast with the surrounding TiO_2_ surface in wettability as in roughness (*Ra*_*PTFE*_ ≈ 3 µm vs. $R{a}_{Ti{O}_{2}}$ ≈ 0.3 µm). See the Methods section for more information. To ensure repeatability, two experimental runs were performed for each boiling setup.

As shown in Fig. 2b, independent of the degassing scheme, boiling on the heterogeneous patterned surface commenced when small bubbles first emerged along the edges of the PTFE spots, followed by quick vapour coalescence that spread over the entire hydrophobic subdomains. Notwithstanding the similar early stages of bubble formation, the actual ONB jumped from an average negative Δ*T*_*sat*_ ≈ −1.5 K in the open system to Δ*T*_*sat*_ ≈ 3.75 K in the closed system, suggesting considerably different levels of gas presence. Specifically, in accordance with the extended bubble nucleation model^9, 31^, the minimal superheat needed for actual bubble growth without collapsing in the presence of dissolved gas should be

1 $${\rm{\Delta }}{T}_{sat}=\frac{T{v}_{fg}}{{h}_{fg}}(\frac{2\sigma }{r}-{P}_{g})$$

where *v*_*fg*_ denotes the specific volume change during phase transition, *h*_*fg*_ is the specific latent heat, and *σ* is the surface tension between the liquid and vapour phases. In their seminal work on subcooled flow boiling of fluorocarbons, Murphy and Bergles^35^ posited that sufficiently high gas concentrations may lower the onset of boiling to “a value below the normal boiling temperature”. Assuming the cavity mouth radius *r* < 3 μm (about the feature size gleaned from the SEM image of the PTFE surface shown in Fig. 2a), we calculate that the gas partial pressure, *P*_*g*_, within the bubble embryo must be at least 0.4 atm to permit negative-superheat nucleation, which is comparable with previous estimates regarding extremely gassy bubble nucleation^31, 32^. It is noteworthy that all previous attempts at degassing non-wetting surfaces failed to adequately eliminate the effect of incondensables on pool boiling since the incipience boiling superheat still fell stubbornly below zero^31^. Hence, it is for the first time, to the best of the authors’ knowledge, that the generation and growth of pure vapour bubbles on a biphilic surface have been observed.

The remarkable disparity in ONB leads to equally different BHT. In sharp contrast to the copper surface, the boiling curves of the biphilic surface exhibit distinctive gaps between the open and closed systems (Fig. 2c). Despite the nearly perfect convergence during natural convection^36^ (at low heat fluxes) and later in the fully developed nucleate boiling regime (at high heat fluxes)^37^, heat transfer in the open system appeared to gain extra boost in the intermediate heat-flux range thanks to a particularly strong Marangoni effect^38, 39^, which is reminiscent of the boiling behaviour of highly subcooled gassy water^40^. Such dissolved gas-induced thermocapillary flow will be explored further in the following section. See Supplementary Note for detailed analyses of the evolution of the heat transfer characteristics.

### To depart or not to depart

**Figure 3:**
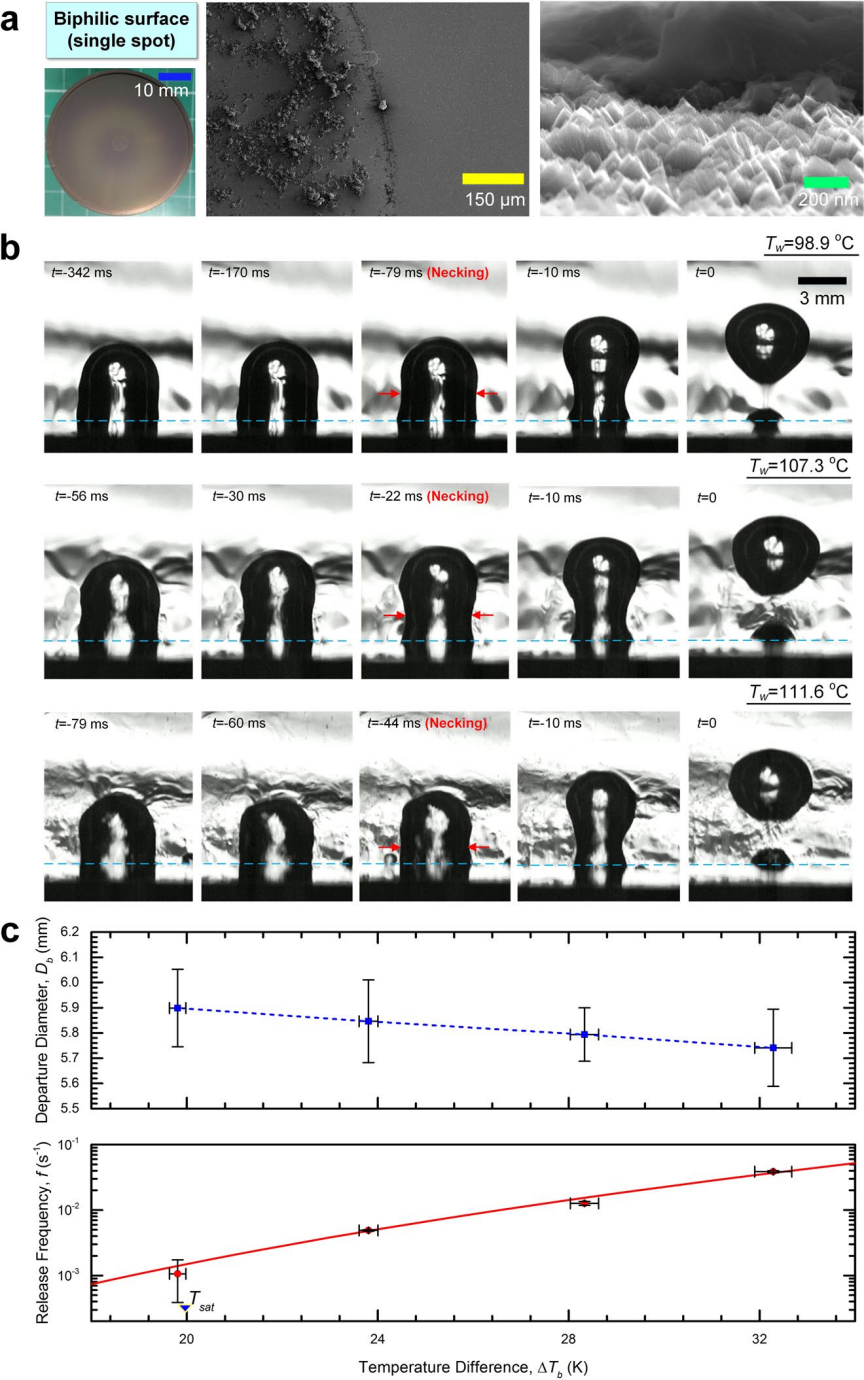
Bubble behaviour in the open system. (**a**) A heterogeneous surface with a single artificial nucleation site. On the superhydrophilic TiO_2_ surface (*θ*_*e*_ ≈ 0°, post UV-irradiation) was deposited by drop-coating a hydrophobic island (*θ*_*e*_ > 145°) with a diameter of 6 mm that was composed of APTES (3-aminopropyltriethoxysilane)-modified halloysite nanotubes and synthesized polymer P(FA-C_8_-*co*-DOPAm). The SEM images confirms dissimilar topography for the non-wetting surface, which was particularly favourable to bubble nucleation. An isolated bubble could preferentially form and grow without interference from neighbouring bubbles. (**b**) Series of high-speed photos showing the process of bubble pinch-off from the biphilic surface at various surface temperatures. The time sequence was set to 0 at the moment of bubble pinch-off. Regardless of the surface temperature, the bubble departure commenced with “necking”. Further stretching of the bubble stem ultimately led to rupture of the interface (with only part of the bubble detached from the surface). The light blue dash line represents the boiling surface. (**c**) Plot of the bubble departure diameter (taken as the equilibrium bubble size before necking), *D*_*b*_, and the release frequency, *f*, versus the excess surface temperature relative to the bulk temperature, Δ*T*_*b*_, based on the analysis of the high-speed imaging results. The bubble dynamics on the biphilic surface cannot be adequately explained by the existing correlations as the observed partial bubble departures involve essentially no waiting time. The red line represents a best fit to the data, *f*~Δ*T*_*b*_^6.70±0.35^. Each data point in the figure is averaged over five consecutive bubble growth periods, with the error bars indicating the spread of the individual measurements.

The different boiling performances between the open and closed systems translate to contrasting bubble dynamics as well, which were examined in more details separately using a heterogeneous surface equipped with a single artificial site of bubble nucleation. Specifically, on the TiO_2_-coated heat transfer surface was deposited a 6-mm circular hydrophobic polymeric coating (*θ*_*e*_ > 145°, containing halloysite nanoparticles) such that an isolated primary bubble can form and grow with minimum thermal interference from neighbouring bubbles (Fig. 3a). See the Methods section for more details with respect to the surface preparation procedures.

Figure 3b shows the time-lapse images of the bubble detachment process under various surface temperatures in the open system. (Complete movies can be found in Supplementary Videos M1) It becomes evident that bubble behaviour hardly differed between the conditions below and above the nominal saturation temperature, *T*_*sat*_ = 100 °C: with its horizontal expansion largely constrained to the end the hydrophobic subregion, the bubble gradually grew into an elongated slug shape. Under the subcooled condition (*T*_*bulk*_ = 80 °C), the consequently sporadic bubble growth eventually led to concavity in the bubble interface, which then turned into an increasingly narrow neck (i.e., so-called “necking”). Partial bubble departure occurred when the above-neck portion of the bubble took off, leaving the bubble base firmly anchored to the hydrophobic island^21, 37^. Figure 3c shows the plot of the distribution of the departure diameter (taken to be the vertical equilibrium size of the bubble prior to necking), *D*_*b*_, along with the corresponding release frequency, *f*, over different surface temperatures. Remarkably, for the moderate decrease in *D*_*b*_, *f* is found to vary by almost two orders of magnitude with temperature, scaled as $f\backsim \,{\rm{\Delta }}{T}_{b}^{6.70\pm 0.35}$, which deviates significantly from the usually strong *f*-*D*_*b*_ correlation predicted by, for example, Zuber’s relation^37^ (with *g* being the acceleration due to gravity, and *ρ*_*l*_ and *ρ*_*v*_ the liquid and vapour densities, respectively)

2 $$f{D}_{b}=0.59\,{[\frac{\sigma g({\rho }_{l}-{\rho }_{v})}{{\rho }_{l}^{2}}]}^{0.25}$$

**Figure 4:**
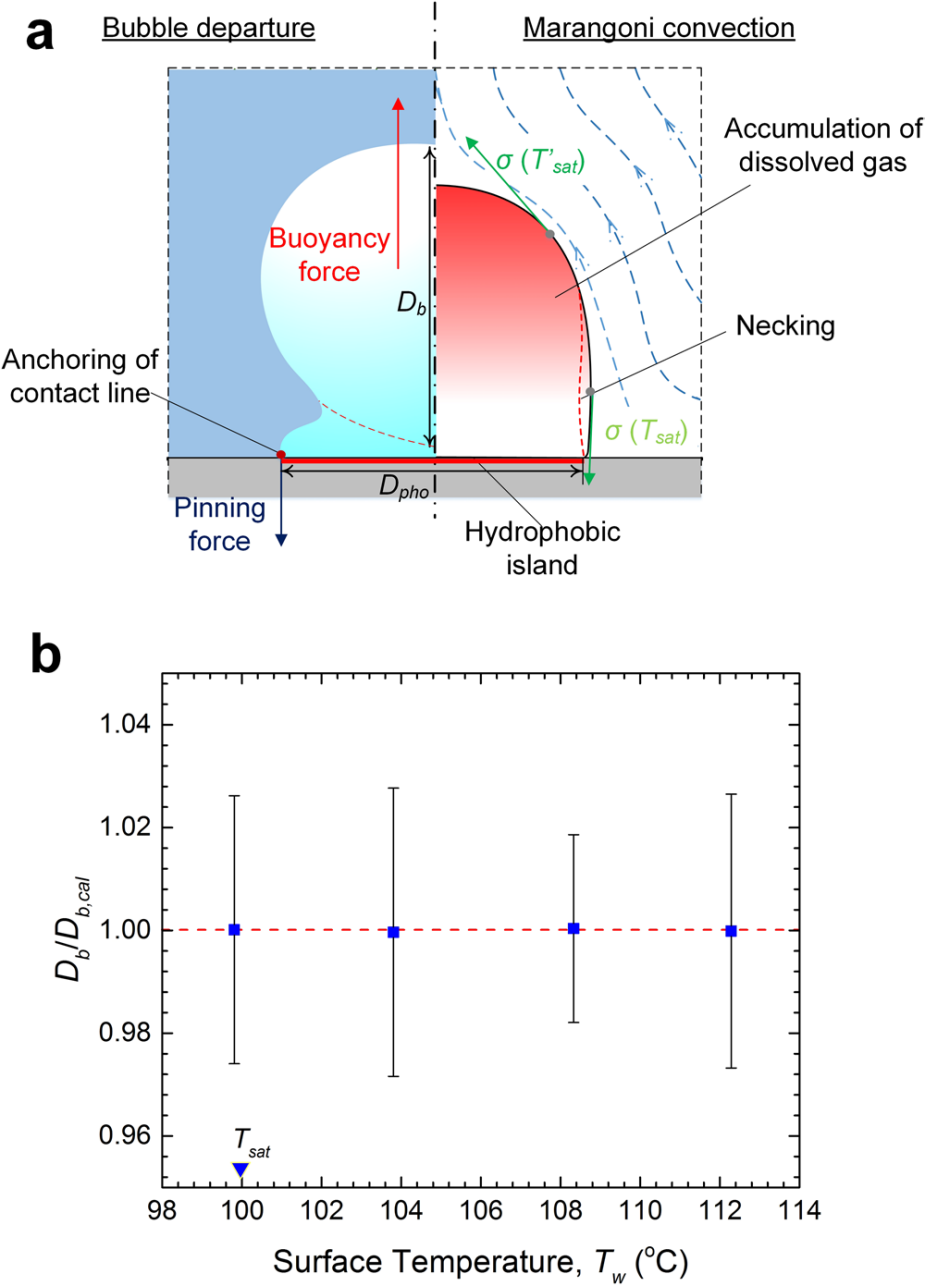
Bubble departure mechanism. (**a**) Surrounded by subcooled liquid, the stationary bubble sitting on the hydrophobic surface assumes an elongated slug shape, as the three-phase contact line is pinned at the end of the non-wetting domain. The increasing buoyancy force results in continuous thinning and stretching of the neck section of the bubble, which leads ultimately to its rupture. Dissolved gas in the water is gasified during evaporation, and ends up being entrained into the bubble by the incoming vapour. The accumulation of gas results in a significant temperature difference between the gas-depleted bottom and gas-rich top of the bubble. The surface stress imbalance contributes to strong thermocapillary effect. (**b**) Plot of the experimentally measured bubble departure diameter, *D*_*b*_, versus the calculations by equation (4), *D*_*b,cal*_. The cofactor in the present empirical correlation, *D*_*b,cal*_ = Λ(*x*_*g*_)${L}_{c}^{^{\prime} }$, depends on the mole fraction of gas within the bubble, Λ(*x*_*g*_) = (0.944 ± 7.398 × 10^−4^) + (0.0648 ± 2.590 × 10^−3^) × *x*_*g*_. Here ${L}_{c}^{^{\prime} }$ = [6*D*_*pho*_ *σ*/*g*(*ρ*_*l*_ − *ρ*_*v*_)]^1/3^ is the modified characteristic capillary length. Error bar: spread of individual measurements over five consecutive bubble growth periods.

In the following, we propose a simple model for (partial) bubble departure from a biphilic surface with high gas contents (Fig. 4a). Traditionally, derived from a balance between the driving buoyancy force and impeding surface tension force (omitting contributions like inertial and drag forces), the bubble size at departure can be determined based on the capillary length, *L*_*c*_ = [*σ*/*g*(*ρ*_*l*_ − *ρ*_*v*_)]^0.5^, leading to semi-analytical correlations such as Fritz’s relation^41^.

3 $${D}_{b,cal}=0.0208{\theta }_{e}{L}_{c}$$

However, for a biphilic surface, the above equation needs to be modified to take into account the strong pinning of the three-phase contact line at the interface between the hydrophobic and hydrophilic subregions, which reads

4 $${D}_{b,cal}={\rm{\Lambda }}({x}_{g}){L}_{c}^{^{\prime} }={\rm{\Lambda }}({x}_{g}){(\frac{6{D}_{pho}\sigma }{g({\rho }_{l}-{\rho }_{v})})}^{1/3}$$

here *D*_*pho*_ is the diameter of the hydrophobic coating, and the coefficient Λ(*x*_*g*_) is an empirical function based on the average molar fraction of the gas component inside the bubble. Released from the evaporating water at the bubble base (near the contact line), formerly dissolved gas is carried, together with vapour, into the bubble. Slow gas re-dissolution, due to inefficient mass diffusion (Supplementary Note), results in rapid gas buildup within the bubble^38, 39^. Estimates of *x*_*g*_ were indirectly obtained by performing micro-thermocouple measurements of the internal bubble temperatures, which exhibit a linear *T*_*w*_ dependence (Supplementary Fig. S5). The significant presence of gas accounts for the difference between the modified capillary length, ${L}_{c}^{^{\prime} }$, and measured *D*_*b*_, with a best-fit curve, Λ(*x*_*g*_) = (0.944 ± 7.398 × 10^−4^) + (0.0648 ± 2.590 × 10^−3^) × *x*_*g*_. In Fig. 4b, we plot the calculated departure diameter, *D*_*b,cal*_, by virtue of equation (4) against *D*_*b*_, which shows good agreement.

Also, the rising gas-to-vapour ratio along the interface towards the bubble apex—due to the increasing vapour condensation—reduces the saturation temperature, ${T}_{sat}^{^{\prime} }$ < *T*_*sat*_, and in turn locally enlarges the interfacial surface tension, *σ*, according to the Eötvös rule^42^. The resulting surface stress imbalance is capable of inducing a strong thermocapillary reaction^40^, as shown in Fig. 4a. More will be discussed later in comparison with numerical simulations.

**Figure 5:**
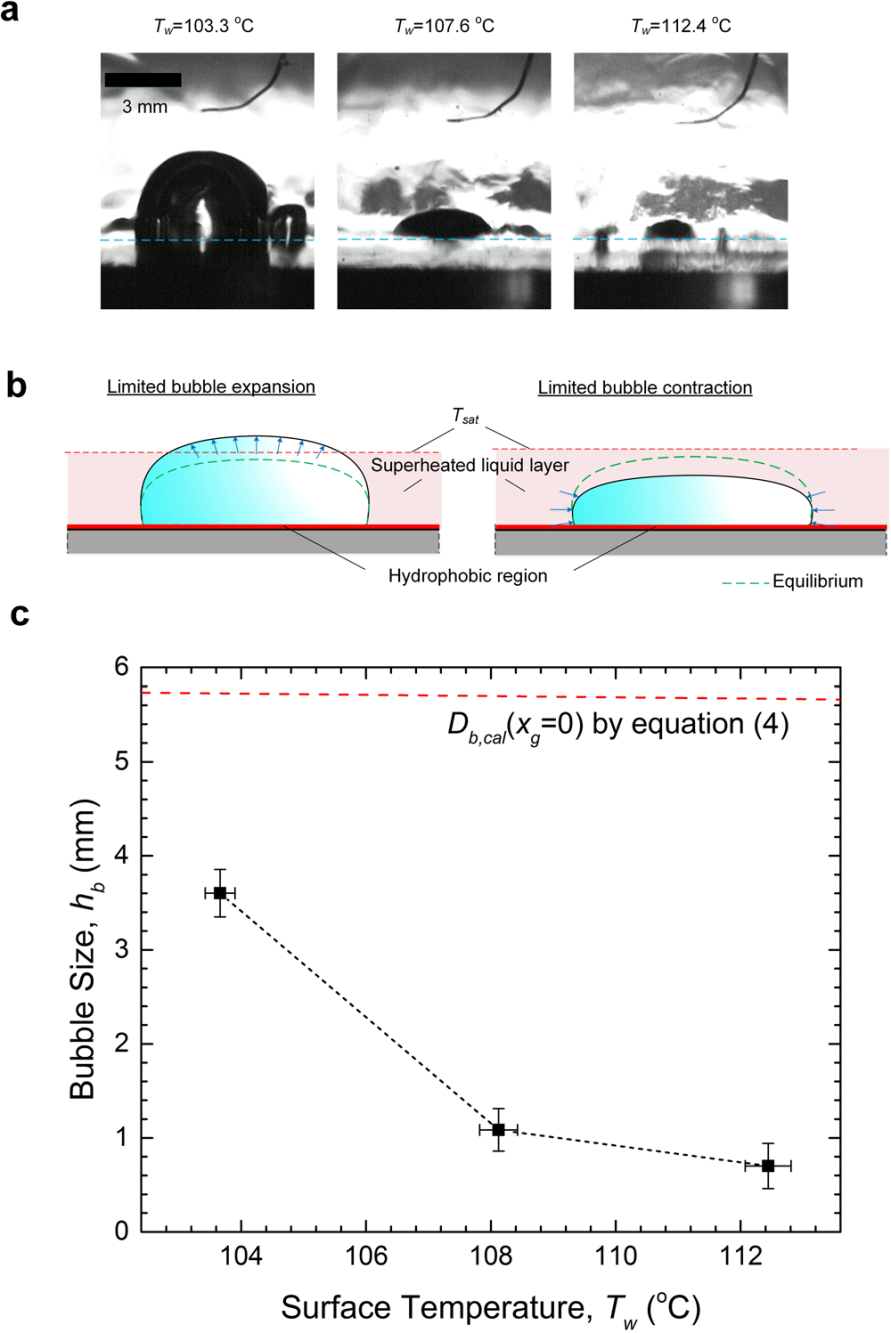
Bubble behaviour in the closed system. (**a**) Evolution of bubble shape with increasing surface temperatures as captured by the high-speed camera. Despite the shrinking size and receding three-phase contact line, the bubble remained immobile on the hydrophobic surface. The light blue dash line represents the boiling surface. See Supplementary Videos M2 for the examples of the oscillatory behaviour of the interface. (**b**) Oscillations of the bubble cap explained by the dynamic equilibrium between evaporation and condensation. The growth of the bubble is vertically confined within a superheated liquid layer above the heat transfer surface. That is because any excess bubble expansion is limited by condensation by the subcooled bulk, and excess contraction by increased evaporation at the base of the bubble. (**c**) Plot of the distribution of the time-averaged (vertical) bubble size, *h*_*b*_, over various *T*_*w*_. The continuous shrinkage of the bubble was for the most part due to the thinning of the superheated liquid layer under increasing surface heat fluxes. For comparison, calculations of the bubble departure diameter by equation (4) in the limit of *x*_*g*_ = 0 are included (red dash-dot line), which shows that without dissolved gas, the bubble never achieved the minimal size needed for the buoyancy force to overcome the hold of the bubble by the surface. The error bars represent the standard deviations of the transient measurements. For detailed transient data, refer to Supplementary Fig. S6.

Free of incondensable impurities, bubble evolution in the closed system followed a fundamentally different path. Besides the considerably delayed boiling inception (at *T*_*w*_ = 103.3 °C, as opposed to *T*_*w*_ = 98.9 °C in the open system), the primary bubble grew to its maximum size shortly after nucleation and remained on the surface without detachment. Moreover, as *T*_*w*_ increased, the steady-state bubble size shrank significantly as the three-phase contact line gradually receded from the edge of the non-wetting area (Fig. 5a). We believe that continuous bubble expansion was hindered by the dynamic competition between evaporation and condensation. Specifically, as shown in Fig. 5b, the bubble tends to contract due to growing condensation once it surpasses the superheated liquid layer and ventures into the highly subcooled bulk; on the other hand, increasing evaporation (relative to condensation) could reverse the bubble shrinkage as it approaches ever closer to the heated surface. As a result, the top of the bubble was found to oscillate up and down around the edge of the superheated layer (see Supplementary Fig. S6 for the transient variations of the bubble size), which becomes increasingly thinner at higher surface temperatures thanks to the cooling effect caused by the enhanced microconvection^43^.

Figure 5c shows the plot of the time-averaged height of the bubble against *T*_*w*_, which includes as reference the calculations of *D*_*b,cal*_ by equation (4) in the extreme of *x*_*g*_ = 0. The results indicate that without incondensables, the bubble simply could not grow to the equilibrium size required for departure from the biphilic surface under the subcooled condition. Note that in the open system, by contrast, the strong gas accumulation inside the bubble resulted in significantly weakened condensation by lessening the driving temperature difference between the bubble and the bulk liquid^44^, such that the bubble was allowed to grow appreciably larger (thus exceeding the pinch-off threshold)^43^.

**Figure 6:**
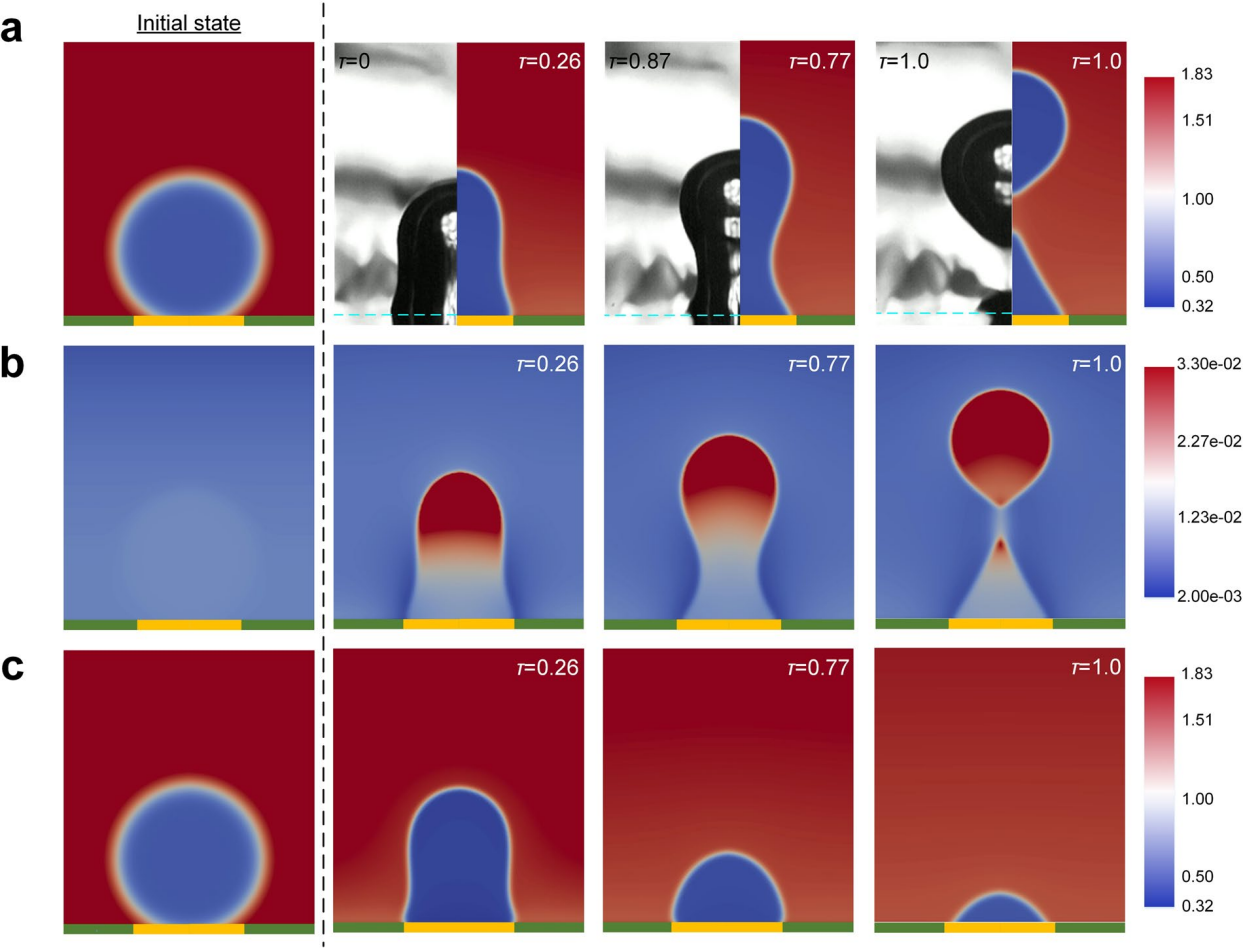
Simulated bubble behaviours with and without the gas presence. (**a**) Bubble pinch-off in a dilute water-nitrogen mixture. The diffuse-interface simulation shows that the vertical bubble deformation leads to the formation of a thinning neck and eventual (partial) departure from the surface. The results compare qualitatively well with the experimental observation at *T*_*w*_ = 98.9 °C in the open case (with the heater surface marked by the green dash lines). The colour scale indicates the total fluid density that is normalized by the critical density of water. Here the nondimensional time, *τ*, is scaled by the bubble departure time (counted from the start of the simulation until the actual instant of bubble pinch-off in the two-component case). For the experiment, on account of the fact that the bubbles largely remain on the surface, the instant *τ* = 0 is set at the moment of “necking” before final departure. Yellow strip: hydrophobic subregion (*θ*_*e*_ = 120°); green strip: hydrophilic subregion (*θ*_*e*_ = 10°). (**b**) Accumulation of incondensable gas within the bubble. The images show the spatial distributions of nitrogen at the same instants as in (a), whose normalized density is represented by the colour scale. Exceptionally high solute concentrations materialize in the upper part of the bubble as the bubble continues to grow on the hydrophobic surface. Consequently, the bubble pinch-off transpires with the ascent of the top of the bubble filled with incondensable nitrogen. Note that the images of the full bubble are produced by merging the axisymmetric simulation results with their mirror counterparts. In Supplementary Videos M3, we include movies depicting the evolutions of the total density and nitrogen density distributions during bubble departure, respectively. (**c**) Shrinkage of a pure vapour bubble. The results show that a continuously shrinking bubble, under the enhanced (contaminant-free) condensation, fails to depart from the surface. In Supplementary Videos M4, we show the bubble growth and eventual collapse in a single-component system.

Corroboration for the profound impact of dissolved gas on bubble behaviour is sought through numerical simulations based on a diffuse-interface method. Built upon the extended van der Waals theory for a binary (two-component) fluid, the hydrodynamic equations for the balances of mass, momentum and energy have been solved by the finite-element numerical toolbox femLego. Details about the physical model, numerical scheme, and computational parameters can be found in the Methods section and Supplementary Note as well as our previous publications^45, 46^. Initially, as shown in Fig. 6a, an axisymmetric half-domain is filled with subcooled water (*T*_*bulk*_ ≈ 0.81 *T* ^*^, *ρ*_*l*_ ≈ 1.89*ρ* ^*^, where *T* ^*^ and *ρ* ^*^ are the critical temperature and density of water, respectively) diluted with a relatively high quantity of nitrogen (*ρ*_*g*_ ≈ 6.6 × 10^−3^ *ρ* ^*^). A saturated spherical vapour bubble (*T* = 0.91 *T* ^*^) is placed on the bottom heating wall (*T* = 0.91 *T* ^*^) that comprises 30% hydrophobic (*θ*_*e*_ = 120°) and 70% hydrophilic (*θ*_*e*_ = 10°) areas.

Bubble expansion first proceeds laterally before reaching the hydrophilic/hydrophobic border, where the three-phase contact line gets pinned. As dissolved gas continues to gather inside the bubble (Fig. 6b), the vertical stretching of the bubble by the growing buoyancy results in bubble thinning (necking) and the eventual pinch-off of the bubble top, in a remarkably similar way to the experiment (see Fig. 6a). It is worth noting that the accumulation of nitrogen (gas) is largely concentrated near the apex of the bubble and weakens considerably approaching the neck section (Fig. 6b). Consequently, the ultimate neck rupture leads incidentally to rough separation of the gas contents from the vapour residuals left behind on the hydrophobic surface. By contrast, with the elimination of the solute nitrogen, the problem is reduced to that of a single-component fluid. As a result of the enhanced impurity-free condensation, the bubble with the same initial size as in the two-component case finds itself affixed to the surface and continuously shrinking under the surrounding liquid after a brief initial period of expansion (Fig. 6c), again in reasonable agreement with the bubble behaviour (namely, lack of bubble departure) in the closed system. The simulated bubble evolutions with and without nitrogen are depicted in Supplementary Videos M3 and M4, respectively.

**Figure 7:**
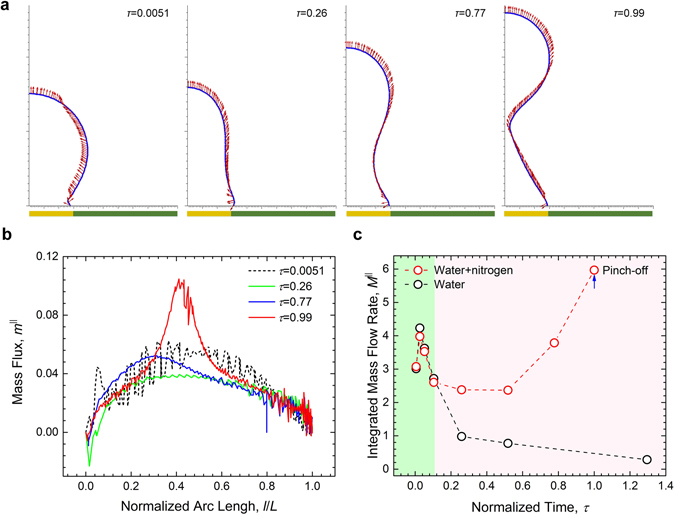
Simulated interfacial flow in the water-nitrogen system. (**a**) Evolution of the local velocity distribution at the bubble interface. The results show that as the bubble grows, the velocity vectors below the neck increasingly align with the bubble surface, which is thought to be driven by the strong surface stress difference between the gas-rich top and vapour-only bottom. Note that the bubble interface is evaluated at the isodensity level of *ρ* = 0.8*ρ*_*l*_. Yellow strip: hydrophobic subregion (*θ*_*e*_ = 120°); green strip: hydrophilic subregion (*θ*_*e*_ = 10°). (**b**) Distributions of the mass flux tangent to the interface, *m* ^||^, over the bubble surface (i.e., the normalized arc length by the total length of the bubble outline) during various stages of bubble growth. Driven by the significant thermocapillary effect, consistently strong Marangoni flow (tangent to the surface) prevails in the lower section of the bubble, which gradually weakens approaching the bubble cap. At the moment of pinch-off, the mass flux rises sharply around the neck region. (**c**) Total tangential mass fluxes integrated over the bubble interface, *M* ^||^, versus nondimensional time, *τ*. Sustained by the ever-increasing surface stress gradient (due to the accumulation of nitrogen inside the bubble), *M* ^||^ strongly diverges nearing the eventual pinch-off. In contrast, in the single-component system, the mass flux along the bubble surface decreases rapidly over time.

The presence of nitrogen can engender a particularly strong thermocapillary effect. In Fig. 7a we illustrate the evolution of interfacial flow surrounding the bubble until its eventual departure from the surface. Note that here the bubble interface is determined at the isodensity contour line, *ρ* = 0.8*ρ*_*l*_. (The choice seems somewhat arbitrary but is deemed justified for the liquid-vapour interface is a continuous transition in the diffuse-interface model and thus has a finite thickness, which precludes an exact definition). There appears to be an increasing trend of the interfacial flow aligning itself with the lower half of the bubble outline (from the base to the neck region, to be precise) towards the moment of pinch-off. The apparent disparity of the nitrogen concentration between the top and bottom of the bubble (recall Fig. 6b) creates a significant surface tension gradient, which gives rise to growing fluid motion along the interface (viz, the Marangoni flow). It should be also noted that such thermocapillary effect seems to be appreciably weaker in the upper half of the bubble, overshadowed by the condensation (represented by flow across the bubble interface). As a result, no rising jet flow^43^ can be detected shooting upwards from the cap of the bubble. One possible explanation might be that the saturation of solute nitrogen within the bubble neutralizes the Marangoni effect by opposing further increases of the interfacial surface tension (on account of the negative dependence of *σ* on the gas concentration).

Based on the results in Fig. 7a, we calculate the tangential mass flux per unit length, ${m}^{||}=\rho \vec{{\bf{v}}}\cdot \vec{{\bf{t}}}$, where $\vec{{\bf{v}}}$ is the velocity vector and $\vec{{\bf{t}}}$ is the unit vector tangent to the bubble surface (pointing towards the top of the bubble). Figure 7b shows the distribution of *m* ^||^ along the arc length (measured from the bottom) of the bubble surface, *l*, that is normalized by the total curve length of the interface, *L*. It can be seen that consistently strong interfacial flow dominates the lower half of the bubble as *m* ^||^ trends upwards before peaking near the midsection of the bubble. Then *m* ^||^ gradually declines and eventually succumbs to large fluctuations towards the bubble cap, which is indicative of the growing instabilities under the increasing condensation and declining thermocapillary effect. Note that at the pinch-off, *m* ^||^ strongly diverges in the neck region. This extraordinary behaviour could be attributed to the intense local temperature variations caused by the rapid contractions of the bubble stem. Figure 7c shows the time-history of the total mass flux integrated over the bubble surface, ${M}^{||}={\int }_{L}{m}^{||}dl$. For comparison, we also include the results of the single-component (water) system. Divergent behaviours arise between these two cases: in the absence of nitrogen, instead of surging as a result of the growing surface stress gradient, the interfacial flow falls sharply after the initial upswing—which is likely to be caused by the entrainment effect accompanying the bubble expansion on the hydrophobic surface—and never recover. Such contrasting behaviours lend support to the importance of dissolved gas in generating and sustaining thermocapillary flows.

## Discussion

As we have seen in the previous sections, unlike the case of the hydrophilic copper surface, subcooled boiling on a biphilic surface differed dramatically between the open and closed systems. The simulations suggest that the fundamental difference in bubble behaviour be attributable to a formidable presence of dissolved gas (*ρ*_*g*_/*ρ*_*l*_ ≈ 3500 ppm). This is somewhat perplexing since the water bulk in the experiments is supposed to be nearly depleted of incondensables. One previous experimental study indicated that for any appreciable impact on boiling, a dissolved gas concentration of at least 5.6 × 10^−3^ mole/mole, or around 480 ppm (in a highly-wetting liquid such as FC-72), is needed^33^. The negligible dissolved gas level following the boiling deaeration in the open system (<5.53 ppm) and vacuum degasification in the closed system (<0.7 ppm) were simply insufficient to engender any meaningful effects on bubble nucleation and/or BHT. Such a seeming paradox raises an interesting question: where does the excess gas originate? One plausible explanation for the gas source has to do with the strong gas enrichment found on hydrophobic surfaces. Recent molecular dynamics simulations^47, 48^ have revealed that due to a slowdown of gas diffusion, a significant amount of gas particles appears to accumulate at sufficiently hydrophobic surfaces that are immersed in water, with concentration levels ranging from ten- to hundred-fold that in the bulk. The considerably increased gas density is suspected to give rise to surface nanobubbles, which have, besides exhibiting a surprising stability against potentially tremendous gas outflow (on account of the tiny bubble size), generated speculations as alternative nucleation sites to vapour-capturing surface cavities^26^.

**Figure 8:**
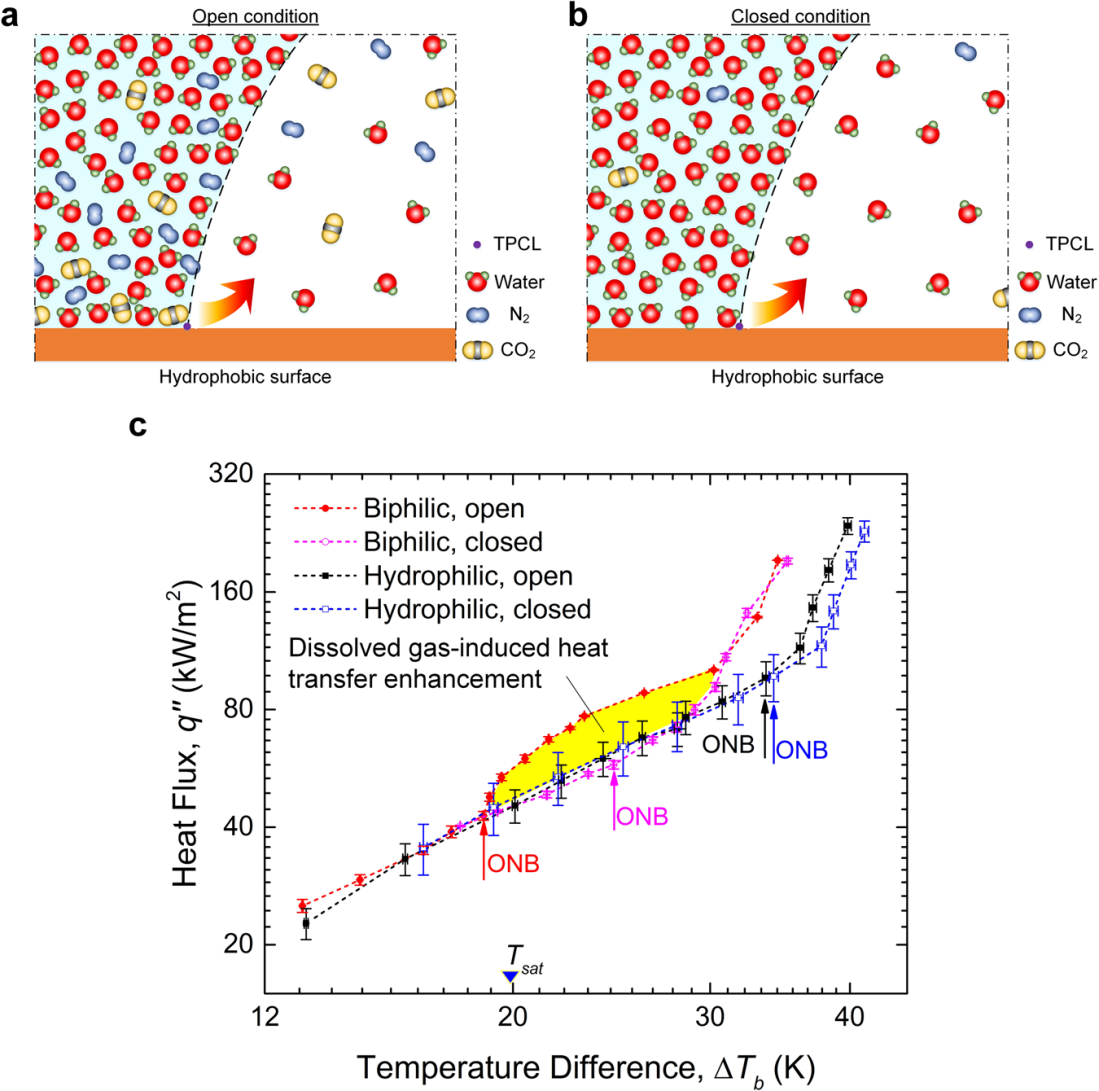
Different mechanisms of bubble generation on a hydrophobic surface and their impact on heat transfer. (**a**) Bubble nucleation in the open system. A considerable amount of gas is believed to aggregate near the hydrophobic surface even when the bulk liquid is nearly depleted of incondensables following boiling deaeration. The liquid phase with such high concentrations of dissolved air tends to become metastable at significantly lower superheats, which allows a mixture of vapour and gas contents to be liberated through the three-phase contact line (TPCL) into the growing bubble. (**b**) Bubble nucleation in the closed system. Under reduced external pressures, the gas enrichment tends to form larger nanobubbles on the hydrophobic surface, the constant merging of which facilitates eventual removal. As a result of the less formidable presence of dissolved gas, vapour bubble nucleation follows the conventional route irrespective of the surface hydrophobicity. **(c)** Comparison of the boiling curves of the biphilic (PTFE/TiO_2_) surface under the open and closed conditions and that of the uniformly superhydrophilic (TiO_2_) surface under similar conditions. The boiling incipience occurred on the biphilic surface at Δ*T*_*sat*_ = −1.6 K in the open system and Δ*T*_*sat*_ = 2.9 K in the closed system, respectively; on the other hand, ONB on the hydrophilic surface was measured to be Δ*T*_*sat*_ = 13.9 K in the open system and Δ*T*_*sat*_ = 14.2 K in the closed system, respectively. It would appear that compared with the very different boiling curves of the biphilic surface, the results of the TiO_2_ surface varied little between the open and closed conditions, denoting little effect of dissolved gas. The significant gap between the boiling curves of the open and closed systems of the biphilic surface (shaded by yellow) represents the impact of the gas accumulation on heat transfer in the low-superheat region.

We hypothesize that such an extraordinary gas presence could react very differently to the boiling deaeration and vacuum degasification, which then leads to divergent paths to bubble nucleation. In the open system, as the bulk water is heated up, the air solubility drops precipitously and the water becomes supersaturated. Aggregations of excess gas molecules are retained by the hydrophobic surface, most likely in the form of nanobubbles or nanopancakes (which proved to be incredibly stable even surviving temperature increases up to the boiling point^49^). Primed for boiling nucleation, these gaseous domains raise much lower energy barriers than vapour-filled cavities^30^. Consequently, both water vapour and gas molecules can be readily pulled from the liquid phase into the activated bubble embryos (Fig. 8a), leading to extremely low-superheat boiling incipience. In the closed system, on the other hand, it is the sudden reductions of the system pressure that triggers supersaturation (as suggested by Henry’s law), but it fails to produce similar air enrichment above the surface. That is because the resulting nanobubbles can be driven from the surface by the enlarged buoyancy force as they tend to grow bigger and merge with each other under vacuum conditions^50^. As a result, against a backdrop made nearly exclusively of water (Fig. 8b), the initiation of boiling on the hydrophobic surface hews more closely to the conventional nucleation of pure vapour bubbles, namely, occurring at positive surface superheats.

A few remarks in regard to the limitations of the nanobubble theory are in order. The considerable gas enrichment seems to occur only in the immediate vicinity of the hydrophobic surface (the height of nanobubbles being a few nanometres), beyond which the extremely high gas density quickly drops to the bulk level^47^. The apparent short-ranginess of the hydrophobic attraction significantly constrains the capacity of the gas reservoir to hold gas molecules, which could not fully account for the high gas content (nearly 40%) in a millimetre-sized bubble growing at negative superheats. Further complicating the matter, as mentioned above, a majority of the accumulated gas, according to the simulations, appears to leave the surface along with the departing bubble. It still remains unclear how the gas supply at the hydrophobic surface can be replenished fast enough to sustain periodic cycles of bubble growth.

This paper has demonstrated that the unique hydrophobicity-induced gassy boiling has a profound impact on BHT on a biphilic surface. In Fig. 8c, we re-plot the boiling curves obtained for the TiO_2_/PTFE surface with and without dissolved air and include, for comparison purposes, the results of the boiling measurements using an uncoated TiO_2_ surface under the same conditions. It can be seen that by depositing an array of hydrophobic PTFE spots, significant heat transfer enhancement has been achieved: the boiling curve exhibits a clear left-hand shift, with ONB falling below zero. It is interesting to note that the boiling curve of the biphilic surface in the closed system settles nicely between those two, which prompts a more nuanced view of the BHT enhancement using the biphilic surface.

After eliminating the effect of dissolved air, nearly all the heat transfer gains in the low-superheat region seems to have vanished. Not only is the boiling incipience significantly delayed (from Δ*T*_*sat*_ = −1.6 K to about Δ*T*_*sat*_ = 2.9 K), but, even with the bubble nucleation on the hydrophobic subregions well underway, there appears to be little immediate noticeable effect on heat transfer following bubble nucleation without gas. The growth of the vapour-gas mixture bubble, as indicated by the diffuse-interface simulations, generates strong thermocapillary flow, which is believed to be responsible for the noticeable heat transfer gains over the closed-condition curve. Not until the boiling enters later stages—where dissolved gas as fuel for extremely gassy boiling is expected to be exhausted^29^—does the impact related to the actual biphilic nature of the surface begin to dominate. That is, more ordered bubble dynamics resulting from the spatially alternating hydrophilic/hydrophobic pattern facilitates efficient vapour removal and liquid replenishment, which leads to higher heat transfer rates than those on the homogeneous TiO_2_ surface (both in the open and closed systems). The insights gained in this study are of particular importance to next-generation thermal management solutions for electronic devices, which requires a quick activation of the more efficient BHT at superheating levels as low as possible. The use of biphilic surfaces has been shown to greatly lower nucleation threshold and effectively expand the nucleate boiling regime.

## Methods

### Surface fabrication and characterization

The heat transfer block was composed of a copper cylinder 30 mm in diameter and 100 mm in length with a tapered lower end for mounting (see Supplementary Fig. S1a). The top of the block was fitted with a 50-mm-wide and 0.3-mm-thick annular “skirt” flush with the block (boiling) surface so that undesirable bubble generation can be suppressed around the edge of the surface. As a reference for pool boiling heat transfer, a plain copper surface (with a static contact angle of 80°) was polished to a mirror finish using a free-abrasive lapping process with an Alumina polishing suspension (0.3-μm grade). To make a superhydrophilic substrate, a thin layer of TiO_2_ with a thickness of about 250 nm was first deposited on a copper surface by the sputtering process, which became superhydrophilic (the contact angle dropping close to 0°) subsequent to continuous UV irradiation for over 12 hours. Two different types of hydrophobic design were prepared for the present study. Specifically, for heat transfer measurements, a total of eight hydrophobic spots (4 × 2 array, patterned using a mask made of adhesive tape) with a 6-mm diameter and 1-mm spacing were made by spray-coating Polytetrafluoroethylene (or PTFE) onto the TiO_2_ substrate. The coatings were then baked in a muffle furnace at 260 °C for 30 minutes. The equilibrium contact angle was measured to be about 120°.

For visualization of bubble dynamics, a single hydrophobic (contact angle around 145°) spot of 6 mm in diameter, patterned by applying a mask, was made on the TiO_2_ substrate by coating of a solution containing polymer/halloysite nanotube composites. Dried halloysite nanotube powder (2 g, 30–70 nm in diameter and 200 nm–2 µm in length, Sigma-Aldrich), along with 1-mL 3-aminopropyltriethoxysilane (or APTES, Shin-Etsu Chemical) was dispersed in 50-mL dehydrated ethanol. The dispersion was heated up to 80 °C and stirred for 8 hours, whose product was then collected by centrifugation. After serval rounds of redispersion with ethanol and centrifugation and being dried at 120 °C in vacuum for 2 hours, the APTES modified halloysite nanotubes were dispersed in 8-mL Asahiklin 225 (or AK225, Asahi Glass). Into the dispersion was added 100-mg P(FA-C_8_-*co*-DOPAm), which was the product of radical copolymerization of 2-(Perfluorooctyl)ethyl acrylate (FA-C_8_, 8.1 g, 15.6 mmol, Daikin Industries) and N-(3,4-Dihydroxyphenethyl)acrylamide (DOPAm, 0.25 g, 1.2 mmol) synthesized from dopamine hydrochloride (Sigma-Aldrich). The coating solution of P(FA-C_8_-*co*-DOPAm) modified halloysite nanotubes (at a concentration of about 3.0 wt%) was stirred for 48 hours under room temperature before being drip-coated onto the TiO_2_ substrate.

Contact angle was measured by the static sessile drop method. A 5-µL droplet of pure deionized water was dispensed from a 22-gauge needle (with an inner diameter of 0.38 mm) and deposited onto the sample surface. After a minimum waiting period of 30 seconds, the inherent contact angle was determined based on the equilibrium droplet profile using a goniometer. Multiple measurements were performed to ensure consistency of the results. Detailed surface morphology was characterized using colour 3D laser microscopy and environmental scanning electron microscopy.

### Boiling heat transfer measurements

We used two custom-made boiling setups for boiling experiments, namely, the open system and the closed system. Both systems consisted of a boiling vessel made of Pyrex glass with a diameter of 120 mm and a height of 450 mm, which was affixed to a top flange and enclosed from below by a heating stage. Embedded with two sheath heaters, the heating stage was capable of providing a maximum power of 700 W to the mounted heat transfer block. Deionized water was introduced into the sealed vessel following evacuation of the vessel by using a vacuum pump. In the open system, deaeration was accomplished by continuously boiling the water for 30 minutes with two coil heaters located near the top and bottom ends of the chamber, while leaving the vessel open to the atmosphere. In the closed system, on the other hand, a two-step degassing procedure was followed: after 1-hour vacuum degasification, the water was first fed into a rubber bellows of 2 L in volume that was encased in an evacuated glass chamber; another hour of vacuum degasification was then applied to the refilled boiling vessel to ensure removal of dissolved gas in the whole setup. Afterwards the system pressure was reverted to the atmospheric level by opening the bellows chamber to the atmosphere. In both systems, to maintain the bulk temperature (monitored by two K-type thermocouples) at 80 °C, an immersed coil heaters and cooler (which was connected to a constant-temperature water bath) were manually adjusted from time to time during the course of the experiment. Heat loss to the surroundings were effectively reduced by using an air heater and foam-board insulation. Three K-type thermocouples with an error of ± 0.2 K (given by the manufacturer) were inserted into the heat transfer block along the centreline at 3 mm, 8 mm, and 13 mm from the boiling surface, respectively. Heat was supplied to the heat transfer block in a stepwise manner. Steady state was assumed to be established when the temperature readings from all three thermocouples fluctuated within ±0.2 K over a minimum interval of 200 seconds. Averaged over 50 consecutive data outputs (at a sampling rate of 5 Hz), the steady-state surface heat flux and the corresponding surface temperature were calculated using Fourier’s law of heat conduction, with maximum fitting errors of 8% and 0.08 K, respectively (see Supplementary Note for details of the uncertainty analysis). Movies of the corresponding boiling process were recorded using a digital video camera. All measurement data were collected by a DAQ system.

### Visualization of bubble dynamics

Bubble dynamics from a single nucleation site on the halloysite nanotube/polymer composite-coated surface were captured using a high-speed camera (v4.3, Phantom, equipped with a Nikkor 180 mm f/2.8D AF lens) at 200–1000 frames/s. At each steady-state surface temperature, the measurements of the bubble departure diameter and frequency (by using a stopwatch) in the open system were averaged over five consecutive cycles of bubble growth and detachment; whereas the quasi-static bubble size in the closed system was determined based on the results obtained over a period of 9–26 seconds (Supplementary Fig. S6). Additionally, the internal bubble temperatures were measured by using a micro-thermocouple^32^, which had an outer diameter of 250 µm and a response time of <15 ms (Supplementary Fig. S5), from which rough estimates of the gas concentrations inside the bubble were derived. The measurement error was about ± 1.5 K based on the specifications provided by the manufacturer.

### Diffuse-interface method simulations

The diffuse-interface method, which is based on the extended van der Waals theory for a two-component system, assumes a finite thickness for the interface, where the surface tension emerges as a result of the continuous transition between the liquid and gas phases. The simulations of the phase-change process of a binary fluid (water mixed with a small amount of dissolved nitrogen) were performed using a finite-element-based symbolic numerical toolbox FemLego^45, 46^, which employed a piecewise linear approximation and first-order Euler forward scheme for spatial and temporal discretization, respectively. The numerical solver was based the characteristics-based split method. Additionally, the interfacial region was resolved by an adaptive mesh refinement. The computational domain had an axisymmetric geometry, which was divided into liquid (bottom) and vapour (top) regions under a thermodynamic equilibrium. To obtain a proper initial condition, we imposed an artificial gravity force as well as a temperate gradient to the saturated system. The resulting steady-state distributions of the temperature and densities provided the background for the boiling simulations, in which a vapour-gas bubble was placed on the heated bottom wall. For the gas-free case, the simulation was carried out with the nitrogen component turned off. More details can be found in Supplementary Note.

## Electronic supplementary material

Supplementary Information

Supplementary Video M1(a)

Supplementary Video M1(b)

Supplementary Video M1(c)

Supplementary Video M2(a)

Supplementary Video M2(b)

Supplementary Video M2(c)

Supplementary Video M3(a)

Supplementary Video M3(b)

Supplementary Video M4
