# Supplementary material for: Early Onset of Nucleate Boiling on Gas-covered Biphilic Surfaces
Source: Sci Rep. 2017 May 17;7:2036. doi: 10.1038/s41598-017-02163-8 (PMC5435692; doi:10.1038/s41598-017-02163-8)
Supplement: Supplementary file 1 — Supplementary Information [file 41598_2017_2163_MOESM1_ESM.doc]

Supplementary Information

Early Onset of Nucleate Boiling on Gas-covered Biphilic Surfaces

Biao Shen^1,*^, Masayuki Yamada^1,2^, Sumitomo Hidaka^2^, Jiewei Liu^3^, Junichiro Shiomi^4^, Gustav Amberg^3^, Minh Do-Quang^3^, Masamichi Kohno^1,2^, Koji Takahashi^1,5^, and Yasuyuki Takata^1,2^

^1^International Institute for Carbon-Neutral Energy Research (WPI-I^2^CNER), Kyushu University, 744 Motooka, Nishi-ku, Fukuoka 819-0395, Japan

^2^Department of Mechanical Engineering, Kyushu University, 744 Motooka, Nishi-ku, Fukuoka 819-0395, Japan

^3^Department of Mechanics, The Royal Institute of Technology, S-100 44, Stockholm, Sweden

^4^Department of Mechanical Engineering, The University of Tokyo, 7-3-1 Hongo, Bunkyo-ku, Tokyo 113-8656, Japan

^5^Department of Aeronautics and Astronautics, Kyushu University, 744 Motooka, Nishi-ku, Fukuoka 819-0395, Japan

*Corresponding author, [shen.biao.604@m.kyushu-u.ac.jp](mailto:shen.biao.604@m.kyushu-u.ac.jp)

Supplementary Note

1. **Timescale of mass diffusion of air in water**

Under the assumption that gas propagation in bulk water is mainly driven by molecular diffusion, we estimate the timescale for the diffusional equilibration of the air-water solution in the open system as

$t_{D}=\frac{L^{2}}{4D_{2}}$ (1-1)

where *L*=150 mm (i.e., the height of the water column above the boiling surface). The mass diffusivity at *T_2_*=80 ^o^C is evaluated using Stokes-Einstein equation,

$D_{2}=\frac{T_{2}}{T_{1}}\frac{\eta_{1}}{\eta_{2}}D_{1}$ (1-2)

where *D_1_*=2.0×10^-5^ cm^2^/s at *T_1_*=25 ^o^C. Here the dynamic viscosities of the solvent (water) are *η_1_*=893.1 μPa s and *η_2_*=354.7 μPa s, which leads to *D_2_*=6.0×10^-5^ cm^2^ s^-1^. Its substitution into equation (1-1) gives *t_D_*=260.4 hours. We note that the process could be considerably accelerated by the effects of natural convection and boiling-induced microconvection.

1. **Uncertainty analysis of the surface temperature and heat flux measurements**

The steady-state boiling surface temperature and heat flux have been extrapolated from the measurements of the three thermocouples embedded in the heat transfer block at *z_1_*=3 mm, *z_2_*=8 mm, and *z_3_*=13 mm relative to the top surface (see Supplementary Fig. S1a). Assuming dominant heat conduction along the *z*-axis (as indicated by the strong linearity of the measured temperature distribution, Supplementary Fig. S1b), we calculate the heat transfer rate in the heat transfer block based on the steady-state one-dimensional heat conduction model,

$q''=\lambda_{c}\frac{dT}{dz}$ (2-1)

where *λ_c_*=394.1 W m^-1^ K^-1^ is the thermal conductivity of copper. Substituting the temperature gradient in the above equation with a least square fit of the thermocouple measurements leads to estimates for the heat flux

$q''=\lambda_{c}\frac{\sum_{i=1}^{3} \left(z_{i}-\bar{z}\right)\left(T_{i}-\bar{T}\right)}{\sum_{i=1}^{3} \left(z_{i}-\bar{z}\right)^{2}}$ (2-2)

and the surface temperature

$T_{w}=\bar{T}-\bar{z}\frac{\sum_{i=1}^{3} \left(z_{i}-\bar{z}\right)\left(T_{i}-\bar{T}\right)}{\sum_{i=1}^{3} \left(z_{i}-\bar{z}\right)^{2}}$ (2-3)

Here $\bar{z}=\sum_{i=1}^{3} z_{i}/3$ and $\bar{T}=\sum_{i=1}^{3} T_{i}/3$ are the mean locations and temperature readings of the thermocouples, respectively. Supplementary Figure S1c and S1d show the collections of the resulting fitting errors (standard deviations) for the surface heat flux, *q*ʺ, and temperature, *T_w_*, respectively, which are found to mostly reside within 8% for *q*ʺ and 0.08 K for *T_w_*.

1. **Characteristics of the boiling curves the open and the closed systems**

Prior to the onset of boiling, heat transfer was dominated by natural convection in both cases, which can be fit to a classical scaling relation^1^, *Nu*~*Ra*^1/3^ (Supplementary Fig. S2). Here the nondimensional Nusselt and Rayleigh numbers are defined as *Nu*= *q*ʺ*L*/Δ*T_b_λ_l_* and *Ra*=*gβL*^3^Δ*T_b_*/*D_T_ν*, respectively, where *g* is the acceleration due to gravity, *λ_l_* is the thermal conductivity of the liquid, *β* is the volume expansivity, *D_T_* is the thermal diffusivity, and *ν* is the kinetic viscosity. All the thermophysical properties are evaluated at the mean temperature between *T_w_* and *T_bulk_*. The characteristic length is chosen to be *L*=6 mm (i.e., the hydrophobic pattern size).

As bubbles began to form on the hydrophobic islands in the open system, the boiling curves start to diverge, which notably persists even after boiling commenced in the closed case as well. The discrepancy coincides with significantly different bubble behaviours. Specifically, compared with their counterparts in the closed system, the bubbles in the open system grew noticeably larger (Fig. 2b), evidently as a result of the added gas component. In addition to the “heat pipe” effect (i.e., the coexisting evaporation at the bottom and simultaneous condensation near the cap that facilitate greater heat flow from the heater surface to the subcooled bulk^1^), the heat transfer got an extra boost from the enhanced Marangoni convection in the open case. The dilution of vapour by the gas content within the bubbles causes diminishing local saturation temperatures and hence the formation of a significant temperature gradient along the interface. The resulting surface tension difference (due to its temperature dependency) in turn drives a strong interfacial flow towards the liquid bulk^2,3^, which leads to more convection heat transfer than can be achieved in the closed system. Hence, we fit, with good agreement (Supplementary Fig. S3), the heat transfer data of the open system to an empirical correlation that is based on the Marangoni number^4^, *Ma*=-(*dσ*/*dT*)Δ*T_b_L/η_l_D_T_*. Here *η_l_* is the dynamic viscosity.

With the increasing heat fluxes, the boiling curves for both cases gradually converge. The dominant heat transfer mechanism now depends more on the intense boiling on the TiO_2_ surface than on the PTFE spots. As opposed to the relatively subdued bubble growth on the PTFE surface, clusters of bubbles of much smaller sizes were nucleated on and departed from the superhydrophilic surface with brisk celerity, in accordance with Fritz’s model of bubble dynamics for highly wettable surfaces^5^. We employ Rohsenow’s correlation, *Nu*~*Re*^0.77^, to describe the growing nucleate boiling to a reasonable accuracy, in which the bubble agitation mechanism is analogized to single-phase forced convection^1^ (Supplementary Fig. S4). Note that with the most unstable Taylor wavelength chosen as the characteristic length, *L*=[*σ*/*g*(*ρ_l_*-*ρ_v_*)]^0.5^, the nondimensional Nusselt number is redefined as, *Nu*=*q*ʺ[*σ*/*g*(*ρ_l_*-*ρ_v_*)]^0.5^/Δ*T_b_λ_l_*, and the Reynolds number is given as *Re*=*q*ʺ[*σ*/*g*(*ρ_l_*-*ρ_v_*)]^0.5^/*h_fg_η_l_*. Here *ρ_l_* is the liquid density, and *ρ_v_* is the vapour density. To summarize, heat transfer in the open system underwent, in increasing order of *q*ʺ, three distinct regimes: natural convection, thermocapillary convection, and finally, fully developed nucleate boiling, which is reminiscent of the evolving boiling behaviour of highly subcooled gassy water^4^.

1. **Numerical simulation of bubble dynamics in a binary mixture**

We have employed the diffuse-interface model to derive a qualitative picture of the phase change process in a binary fluid (namely, 1-water; 2-nitrogen). Based on the Helmholtz free-energy functional, the diffuse-interface method^6,7^ assumes a finite thickness for the liquid-gas interface. The surface tension emerges naturally from the continuous transition as a result of incorporating gradient contributions of order parameters (in this case, the densities of the two components, *ρ_1_* and *ρ_2_*) in the energy and entropy formulations. We introduce the following scaling (for the sake of readability, we mark the dimensionless variables with prime ʹ):

$\begin{matrix} r'=L^{*}r,z'=L^{*}z,t'=t^{*}t, \\ \rho'=\rho^{*}\rho,T'=T^{*}T,P'=P^{*}P,e'=\frac{P^{*}}{\rho^{*}}e \end{matrix}$ (4-1)

where the critical parameters (*ρ*^*^=*m_2_*/3*b_2_*, *P*^*^=*a_22_*/27*b_2_^2^*, and *T*^*^=8*a_22_*/27*k_B_b_2_*, with *k_B_* being the Boltzmann constant) of the second component (i.e., water) are chosen as the scales of density, pressure, and temperature, respectively. The characteristic length is *L*^*^=2*b_2_*^1/3^, and the characteristic time is *t*^*^=6*b_2_*^5/6^(*m_2_*/*a_22_*)^1/2^. Here *m_i_* represents the molecular mass of the component i, *a_ii_* offers a measure of the attraction force between component i and component j, and *b_i_* is the molecular volume of component i. The dimensionless balance laws (mass, momentum, and energy) can be written as (omitting ʹ),

$\frac{\partial\rho}{\partial t}+\nabla\cdot\left(\rho\vec{\mathbf{v}}\right)=0$ (4-2)

$\frac{\partial\rho_{1}}{\partial t}+\nabla\cdot\left(\rho_{1}\vec{\mathbf{v}}\right)=\nabla\cdot\left[R_{M}\frac{\rho_{1}\rho_{2}}{\rho^{2}}\nabla\left(\frac{\hat{\mu}_{1}-\hat{\mu}_{2}}{T}\right)\right]$ (4-3)

$\frac{\partial\left(\rho\vec{\mathbf{v}}\right)}{\partial t}+\nabla\cdot\left(\rho\vec{\mathbf{v}}\vec{\mathbf{v}}\right)=R_{P}\nabla\cdot\overleftrightarrow{\hat{\mathbf{P}}}+\mathrm{Re}^{-1}\nabla\cdot\overleftrightarrow{\mathbf{\Gamma}}+R_{G}\rho\vec{\mathbf{e}}_{\mathbf{z}}$ (4-4)

$\begin{matrix} \frac{\partial\left(\rho e\right)}{\partial t}+\nabla\cdot\left(\rho e\vec{\mathbf{v}}\right)=\nabla\cdot\left[\left(\overleftrightarrow{\hat{\mathbf{P}}}+R_{P}^{-1}\mathrm{Re}^{-1}\overleftrightarrow{\mathbf{\Gamma}}\right)\cdot\vec{\mathbf{v}}\right]+R_{T}\nabla\cdot\left[\left(\lambda_{12}\rho_{1}+\rho_{2}\right)\nabla T\right] \\ -\sum_{i,j\in\left\{1,2\right\}} \nabla\cdot\left(\frac{TD_{i,j}}{m_{i}m_{j}}\frac{d\rho_{i}}{dt}\nabla\rho_{j}\right) \end{matrix}$ (4-5)

with the Helmholtz free-energy density

(4-6)

Here the function for the volume fraction occupied by the molecules is defined as φ *φ*=*ρ_2_*/3+*m_21_B_1_ρ_1_*. The nondimensional parameters are *A_11_*=*a_11_*/*a_22_*, *A_12_*=*a_12_*/*a_22_*, *B_1_*=*b_1_*/*b_2_*, *m_21_*=*m_2_*/*m_1_*, and *R_A_*=(1/3)(*b_2_*^1/3^ℏ^2^27π/4*a_22_m_2_*)^3/2^. In the above equations, the generalized chemical potential is given as

$\hat{\mu}_{i}=\mu_{i}-\sum_{j} \frac{TD_{i,j}}{m_{i}m_{j}}\nabla^{2}\rho_{j}$ (4-7)

and the generalized pressure tensor reads

(4-8)

where

(4-9)

and $\overleftrightarrow{\mathbf{I}}$ denotes metric tensor. And the thermodynamic pressure is defined as

$P=\rho_{1}\frac{\partial f\left(\rho_{1},\rho_{2}\right)}{\partial\rho_{1}}+\rho_{2}\frac{\partial f\left(\rho_{1},\rho_{2}\right)}{\partial\rho_{2}}-f\left(\rho_{1},\rho_{2}\right)$ (4-10)

The total energy in equation (4-5) is

$\rho e=\rho u+\frac{1}{2R_{P}}\rho\vec{\mathbf{v}}^{2}$ (4-11)

with the internal energy derived as

$\rho u=f\left(\rho_{1},\rho_{2}\right)-T\frac{\partial f\left(\rho_{1},\rho_{2}\right)}{\partial T}$ (4-12)

In equations (4-4) and (4-5), the viscous stress tensor is given as

$\overleftrightarrow{\mathbf{\Gamma}}=\left(\eta_{12}\rho_{1}+\rho_{2}\right)\left(\nabla\vec{\mathbf{v}}+\nabla\vec{\mathbf{v}}^{\perp}\right)+\frac{1}{3}\left(\eta_{12}\rho_{1}+\rho_{2}\right)\overleftrightarrow{\mathbf{I}}\nabla\cdot\vec{\mathbf{v}}$ (4-13)

where *η_12_*= *η_1_*/*η_2_* is the ratio of the dynamic viscosities for the two components. The rest of the dimensionless parameters include: the Reynolds number, *Re*=*L*^*2^/*t*^*^*η_2_*; the ratio of the attractive potential energy to the molecular kinetic energy, *R_P_*=*P*^*^*t*^*2^/*ρ*^*^*L*^*2^; the ratio of the thermal conductivities for the two components, *λ*_12_=*λ*_1_/*λ*_2_; the normalized mobility coefficient *R_M_*=*P*^*^*t*^*^*M_f_*/*L*^*2^*ρ*^*2^*T*^*^; *R_T_*= *λ*_2_*T*^*^/*ρ*^*^*e*^*^*v*^*^*L*^*^ and *D_i,j_*=*κ_ij_k_B_T*^*^*ρ*^*2^/*m_2_^2^L*^*2^*P*^*^, which relates to the surface tension through the bulk Helmholtz free energy,

$\hat{f}\left(\rho_{1},\rho_{2}\right)=f\left(\rho_{1},\rho_{2}\right)+\frac{1}{2}\sum_{i,j\in\left\{1,2\right\}} \kappa_{ij}\nabla\rho_{i}\cdot\nabla\rho_{j}$ (4-14)

Or, in a more explicit form (with *x* denoting the coordinate normal to the interface),

$\sigma=\sum_{i,j\in\left\{1,2\right\}} \int_{-\infty}^{+\infty} \kappa_{ij}\frac{\partial\rho_{i}}{\partial x}\frac{\partial\rho_{j}}{\partial x}dx$ (4-15)

Here *κ_ij_* (*i*=*j*) denotes the capillary coefficient for the component i and *κ_ij_* (*i*≠*j*) the mixing parameter. We note that the normalized gravitational acceleration is formulated as, *R_G_*=*t*^*2^*g*/*L*^*^. In order to fully resolve the finite interface between the aqueous and gaseous phases (~ tens of angstroms), the physical model is limited to extremely small space (~ hundreds of nanometres) and time scales (~ a few nanoseconds) such that no exorbitant computational costs would be incurred. Consequently, the gravitation acceleration, *g*, needs to be artificially inflated by more than nine orders of magnitude so as to have an appreciable impact on bubble dynamics.

All simulations have been performed using a finite-element numerical toolbox femLego^8^, which employs symbolic computation to solve complex partial differential equations. Specifically, the problem formulations (including governing equations, initial and boundary conditions) were fed into a single MAPLE worksheet, whose compilation then automatically generated complete computer source codes (both in C and FORTRAN). The numerical scheme was based on the characteristic-based split (CBS) method. A piecewise linear approximation and first-order Euler forward scheme were used in space and time discretization, respectively. In addition, adaptive mesh technique was employed to provide a higher resolution in the liquid-gas interfacial region, in which cells with large density gradient were refined locally. The iteration tolerances for all the equations were set to 10^-8^.

We have considered an axisymmetric domain (0.001≤*r*≤100.001, -300.001≤*z*≤-0.001, with the plane of symmetry (*r*=0) removed to avoid potential singularity) filled with a two-phase mixture of water and nitrogen: *ρ_i_^v^*=0.241 (gaseous phase) in the upper one-third of the enclosure; and *ρ_i_^l^*=1.934 (aqueous phase) in the lower two-thirds, at an initial uniform temperature of *T_i_*=0.80. Except at the left boundary where symmetric boundary conditions were applied, all bounding walls were assumed to be no-slip. Both the bottom and top walls were isothermal (Dirichlet), while the right wall was perfectly insulated (Neumann). A dilute presence of nitrogen (*ρ_1i_*=0.01) was evenly distributed everywhere.

First, we obtained the thermodynamic equilibrium state of this binary system under gravity (*R_G_*=5×10^-4^) and a vertical temperature gradient (with the temperature at the bottom wall raised to *T_bot_*=0.82). The relevant parameters were given as follows: *m_21_*=0.8111, *R_A_*=1.332×10^-5^, *B_1_*=1.267, *A_11_*=0.247, *R_M_*=0.170, *D_11_*=0.01, and *D_22_*=1.0. Note that for a single-component fluid, calculations of the surface tension by equation (4-15) prove to be much less sensitive to *D_ii_* approaching the critical point. Following the mixing rule, we calculated *D_12_*=(*D_11_D_22_*)^1/2^=0.1. Also, we chose a non-zero *A_12_*(=0.1), which is a measure of the attractive force between water and nitrogen molecules, so that water and nitrogen can coexist in both aqueous and gaseous states. Furthermore, as *A_12_*<*A_11_*, nitrogen molecules evidently prefer the company of their own kind, which gives rise to slow transport of nitrogen molecules across the interface from the liquid phase to the gas phase.

The initialization led to a mean steady-state density of *ρ_1_^l^*=6.604×10^-3^ in the aqueous phase, as opposed to *ρ_1_^v^*=1.703×10^-2^ in the gaseous phase. Next, on the basis of the obtained initial conditions, we carried out simulations of bubble behaviour in pool boiling with and without the presence of dissolved gas on a computational grid of 41×121 and a uniform timestep of Δ*t*=0.01. A saturated bubble embryo with a radius of *R_b_*=40 and temperature *T_b_*=0.91 mixed with a trace amount of nitrogen gas (*ρ_1_^b^*=0.01) was placed at *r*=0 and *z*=-265, with the temperature at the bottom wall raised to *T_bot_*=0.91. Thus, the Bond number (as a ratio of the gravitational force to the surface tension force) was *Bo_sim_*=4*R_G_*(*ρ^l^*-*ρ^v^*)*R_b_*^2^/*σ*=6.95, which more or less matches that of the experiment, *Bo_ex_*=5.94. The wetting boundary conditions at a solid wall for a binary fluid can be written as,

(4-16)

where $\vec{\mathbf{n}}$ is a unit vector normal to the surface, *θ_e_* is the equilibrium contact angle. With the assumption of contact line motion being independent of dissolved gas*,* equations (4-16) can be simplified to

(4-17)

which is the same as that for a single-component system. Here the nondimensional third-order polynomial function **(*ρ*) was specified as, **(*ρ*)=-1.071*ρ*^3^+3.34*ρ*^2^-2.267*ρ*. A non-uniform *θ_e_* was assumed for the bottom wall (*θ_e_*=120° for *r*<30; *θ_e_*=10° for *r*>30) with the discontinuity at *r*=30 replaced by a smooth transition described by a hyperbolic tangent function, which makes the surface essentially *biphilic*.

References

1. Bejan, A. & Kraus, A. D. *Heat Transfer Handbook* 562-563; 653-654 (Wiley 2003).
2. Marek, R. & Straub, J. The origin of thermocapillary convection in subcooled nucleate pool boiling. *Int. J. Heat and Mass Tran.* **44,** 619–632 (2001).
3. Straub, J. Origin and effect of thermocapillary convection in subcooled boiling. *Ann. N.Y. Acad. Sci.* **974,** 348-363 (2002).
4. Petrovic, S., Robinson, T. & Judd, R. L. Marangoni heat transfer in subcooled nucleate pool boiling. *Int. J. Heat and Mass Tran.* **47,** 5115–5128 (2004).
5. Nam, Y., Wu, J., Warrier, G. & Ju, Y. S. Experimental and numerical study of single bubble dynamics on a hydrophobic surface. *J. Heat Trans.-T. ASME* **131,** 121004 (2009).
6. Liu, J., Do-Quang, M. & Amberg, G. Thermohydrodynamics of boiling in binary compressible fluids. *Phys. Rev. E* **92,** 043017 (2015).
7. Liu, J., Amberg, G. & Do-Quang, M. Diffuse interface method for a compressible binary fluid. *Phys. Rev. E* **93,** 013121 (2016).
8. Amberg, G., Tönhardt, R. & Winkler, C. Finite element simulations using symbolic computing. *Mathematics and Computers in Simulation* **49,** 1–18 (1999).

Supplementary Figures

**
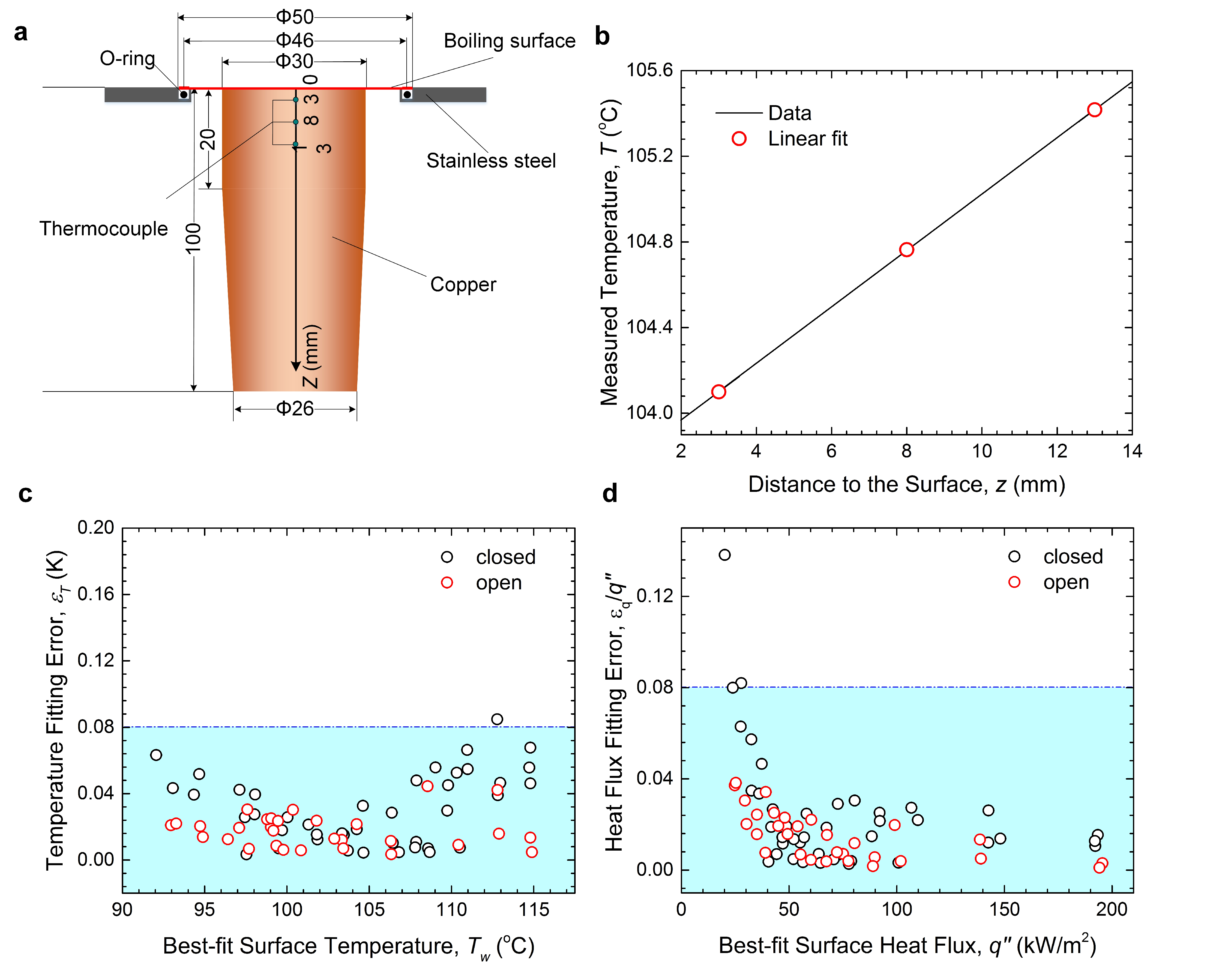
Figure S1** | **Data reductions for the steady-state surface temperature and heat flux. (a)** Schematic for the copper heat transfer block. Three thermocouples, which were embedded in the heat transfer block at *z*=3 mm, 8 mm, and 13 mm from the top surface, respectively, were used to obtain the surface temperature and heat flux. Here the *z*-axis points downward, with *z*=0 residing at the boiling surface. Note that an annular (Φ50-mm and 0.3-mm-thick) “skirt” was welded to the Φ30-mm boiling surface so as to suppress undesired boiling around the edge of the surface. **(b)** Typical temperature measurement results. The steady-state temperature distribution along the heat transfer block shows strong linearity, which lends support to the use of Fourier’s law in estimating the surface temperature and heat flux. The extrapolations lead to fitting errors of about 0.08 K for the surface temperature in **(c)**, and of about 8% for the surface heat flux in **(d)**.


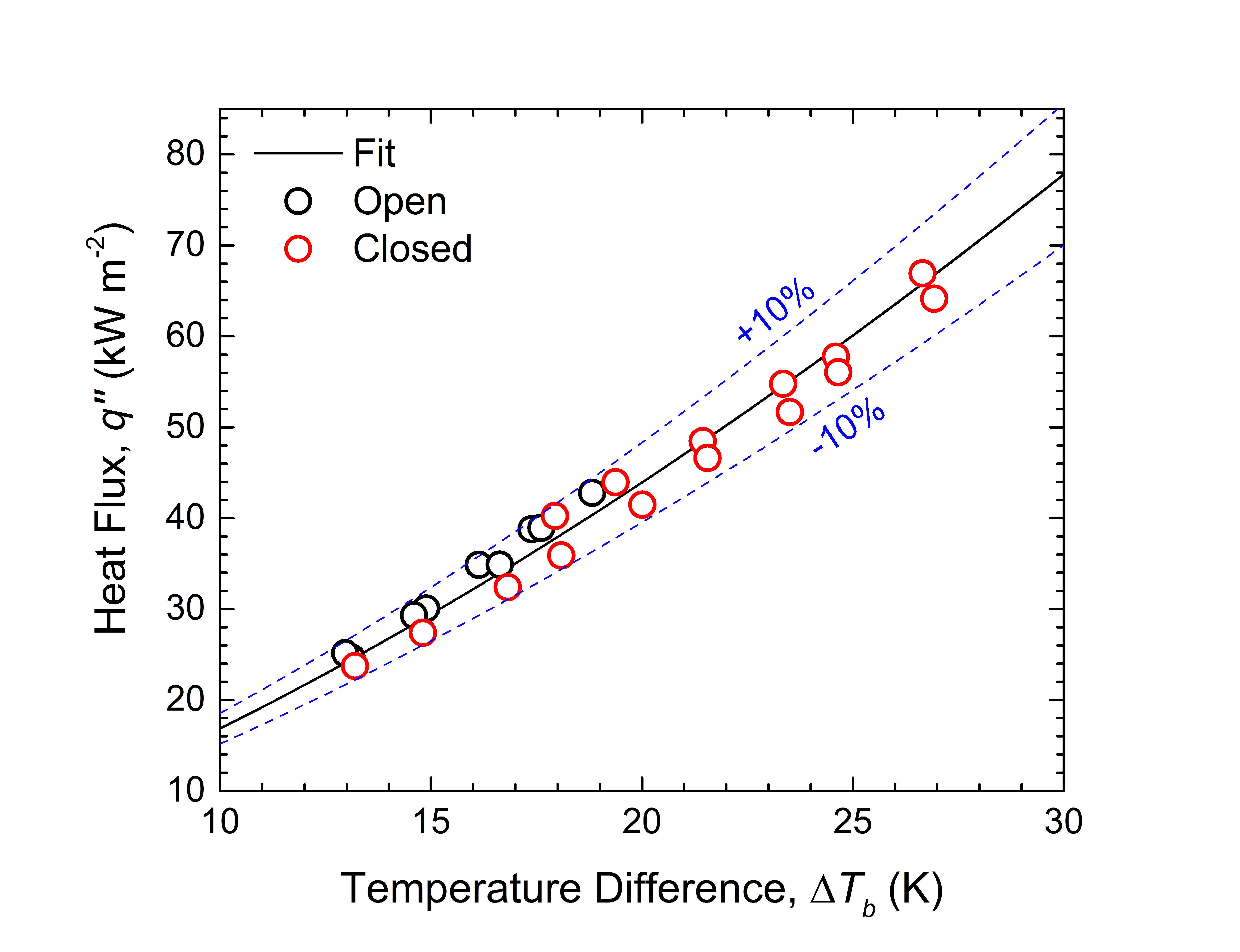


**Figure S2** | **Heat transfer at low surface superheats.** Plot of the applied heat flux, *q”*, versus the temperature difference between the heater surface and the bulk liquid, Δ*T_b_*, of the biphilic (PFFE/TiO_2_) surface in the open and closed systems, respectively. Prior to the boiling incipience, heat transfer was dominated by natural convection. The experimental data can be fit to an empirical correlation^1^, *q”*=*C^NC^*(*gβλ_l_*^3^/*D_T_ν*)^1/3^ Δ*T_b_*^4/3^, where the fitting parameter *C^NC^*=0.239±0.002.

**
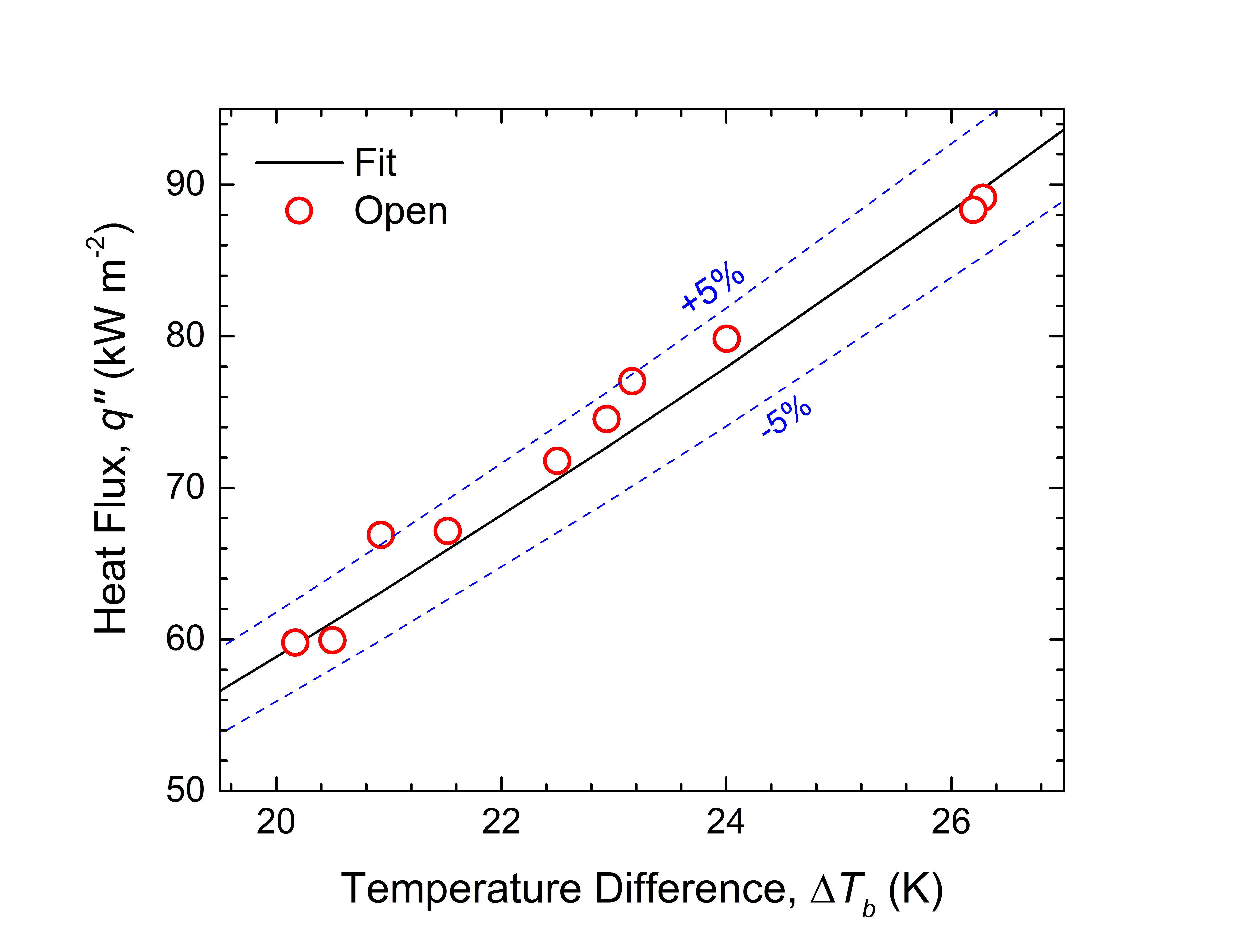
**

**Figure S3** | **Heat transfer at intermediate surface superheats.** Plot of the applied heat flux, *q”*, versus the temperature difference between the heater surface and the bulk liquid, Δ*T_b_*, of the biphilic (PFFE/TiO_2_) surface in the open systems. On each hydrophobic island on the surface sat a large and mostly stationary bubble, which was surrounded by strong surface tension-driven flow due to the dissolved gas-induced temperature gradient along the interface. As a result, the experimental data is fit to an empirical correlation^4^, *q”*=(1+*C^MC^Ma*^0.5^)Δ*T_b_λ_l_*/*L*, which is based on the nondimensional Marangoni number, *Ma*=-(*dσ*/*dT*)Δ*T_b_L/η_l_D_T_*. Here the fitting parameter is *C^MC^*=0.038±5.345×10^-4^.

**
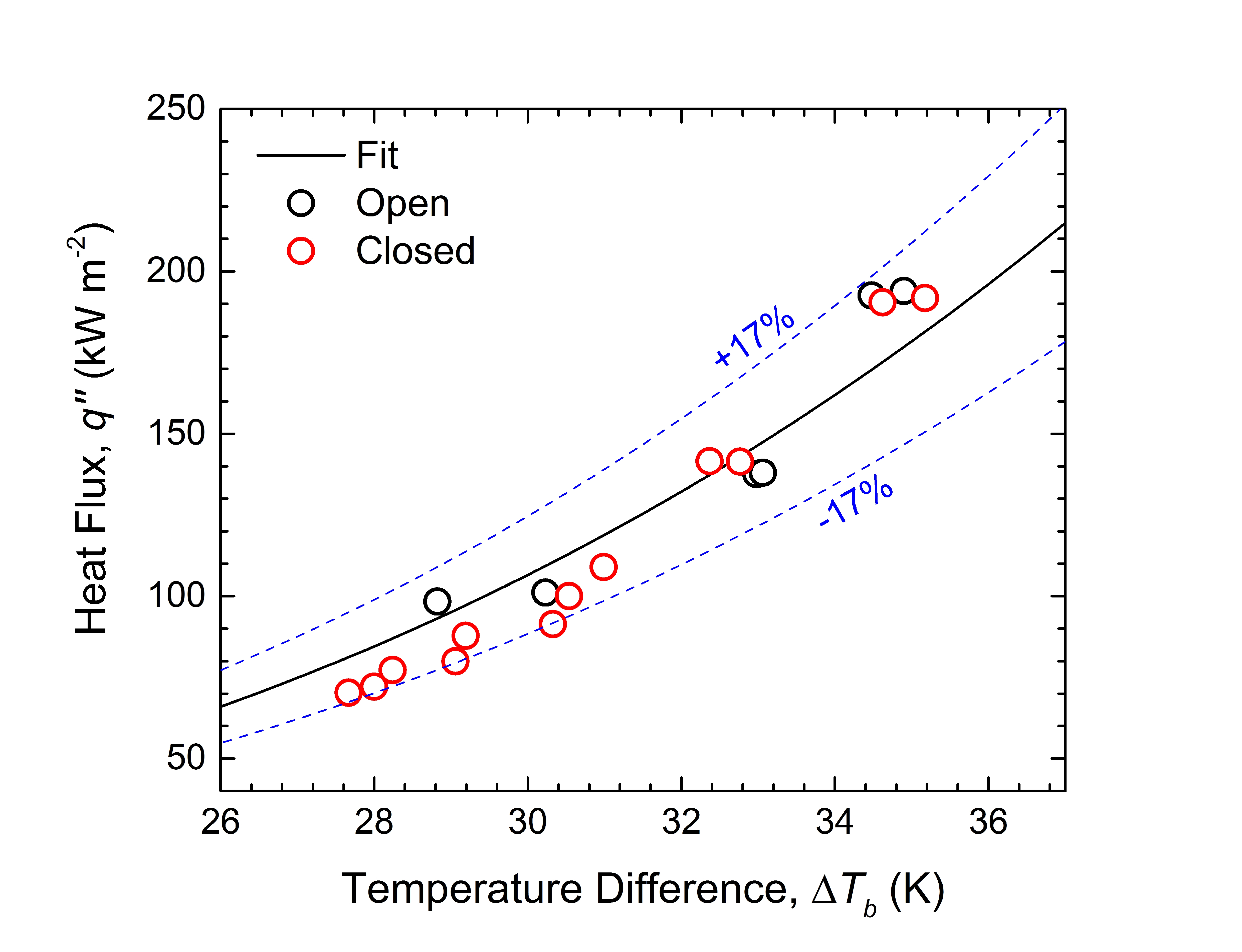
**

**Figure S4** | **Heat transfer at high surface superheats.** Plot of the applied heat flux, *q”*, versus the temperature difference between the heater surface and the bulk liquid, Δ*T_b_*, of the biphilic (PFFE/TiO_2_) surface in the open and closed systems, respectively. The transition to the regime of fully-developed nucleate boiling was accompanied by nucleation of clusters of small bubbles on the TiO_2_ surface and their speedy departure, which is fit to Rohsenow’s well-known empirical correlation^1^, *C_p,l_*Δ*T_b_*/*h_fg_*=*C^NB^*{*q”*[*σ*/*g*(*ρ_l_*-*ρ_v_*)]^0.5^/*h_fg_η_l_*}^0.33^*Pr*. Here *C_p,l_* is the specific heat at constant pressure of the water, and *Pr* is the nondimensional Prandtl number. The fitting parameter is chosen to be *C^NB^*=0.041±3.015×10^-4^.

**
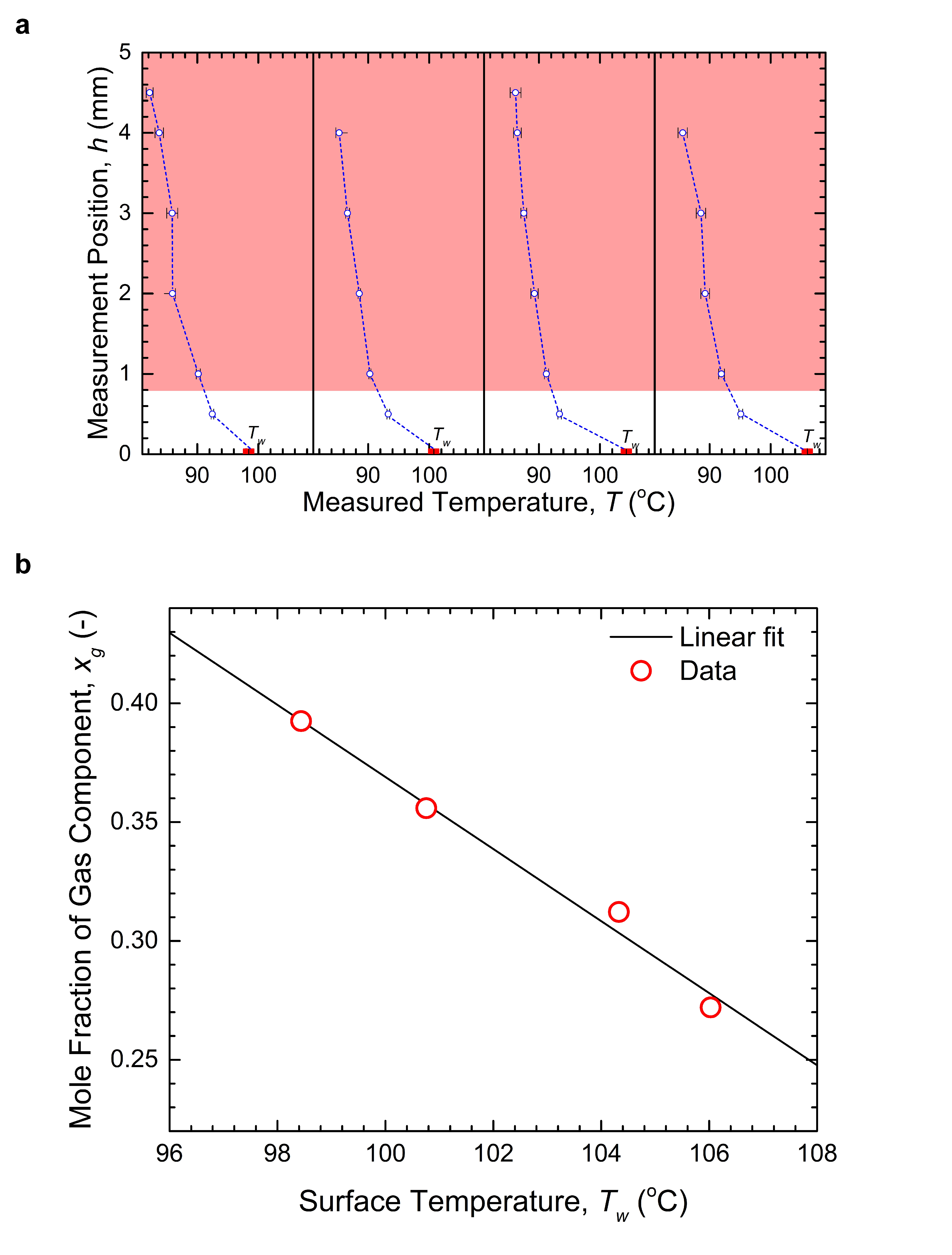
Figure S5** | **Estimates of dissolved gas concentration inside a bubble growing on the hydrophobic subregion in the open system. (a)** Measurements of the bubble temperature at different surface heat fluxes using a Φ250-µm micro-thermocouple. For each bubble growth cycle, a single measurement was performed by placing the micro-thermocouple at a fixed location (1 mm, 2 mm, 3 mm, 4 mm, and 4.5 mm along the centreline of the bubble from the surface). Once the bubble assumed a relatively stable shape, the temperature readings with a sampling rate of 5 Hz were recorded and averaged over a period of 2~5 s to yield quasi-steady state results, which show a continuous decline from the wall. Because of the strong oscillations of the bubble cap, the measurements show an increasing data spread (represented by the error bars) towards the top. In spite of the increasing surface temperature, *T_w_*, the bubble temperature remained persistently below the nominal saturation temperature of 100 ^o^C, which attests to the significant presence of gas inside the bubble. On account of the gas component being accumulated in the upper part of the bubble, we take the mean bubble temperature in the red-shaded area as the apparent saturation temperature, *T’_sat_*, from which rough estimates of the gas amount can be derived. **(b)** Plot of the calculated mole fraction of the gas component inside the bubble, *x_g_*, versus the surface temperature, *T_w_*. Based on the measurements of *T’_sat_* in (a), the gas partial pressure is evaluated as, *P_g_*=*P_t_*-*P’_sat_*(*T’_sat_*)=*x_g_P_t_*, by virtue of Dalton’s law. The obtained distribution of *x_g_* exhibits a linear dependence on *T_w_* in the temperature range considered, which can be fit by *x_g_* =(1.885±0.129)-(0.015±0.001)×*T_w_* (black line).

**
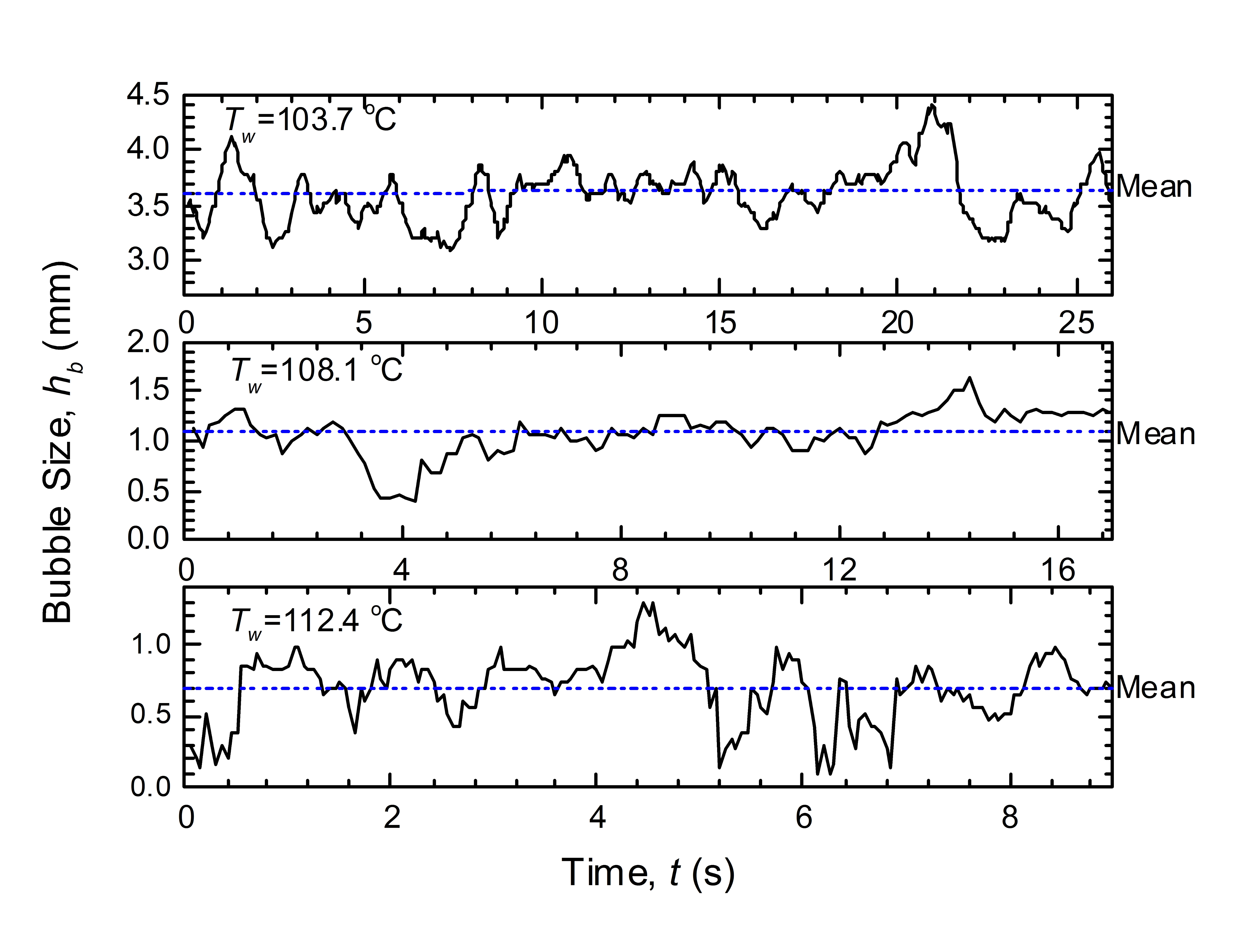
**

**Figure S6** | **Shrinking bubbles in the closed system.** Based on the analysis of the high-speed images taken at an interval of 50 ms, we obtain the transient variations of the (vertical) size of the bubble, *h_b_*, at different surface temperature, *T_w_*. The fluctuations in the data are attributed to the competition between evaporation near the three-phase contact line and condensation by the subcooled bulk. The time-averaged *h_b_* is found to decline precipitously with increasing *T_w_*, which could be explained by the continuous thinning of the superheated liquid layer above the boiling surface thanks to the enhanced heat transfer at high heat fluxes.

Supplementary Video Captions

**Video M1** | **Bubble detachment from the biphilic surface in the open system.** The process of the bubble necking and eventual pinch-off from the surface was captured by high-speed photography at a frame rate of 1000 s^-1^. The surface temperature was (a) *T_w_*=98.9 ^o^C, (b) *T_w_*=107.3 ^o^C, and (c) *T_w_*=111.6 ^o^C, respectively.

**Video M2** | **Oscillations of bubble interface in the closed system.** The unstable bubble expansion and contraction due to the dynamic competition between the effects of evaporation and condensation were captured by high-speed photography at a frame rate of 200 s^-1^. The surface temperature was (a) *T_w_*=103.3 ^o^C, (b) *T_w_*=107.6 ^o^C, and (c) *T_w_*=112.4 ^o^C, respectively.

**Video M3** | **Diffuse-interface simulations of bubble dynamics on a biphilic surface in a two-component system.** (**a**) The evolution of the total density distribution (half-domain) under the influence of an artificially enlarged gravity. The results show in turn the elongation of the vapour-gas bubble, the neck formation, and the ultimate bubble pinch-off. (**b**) The evolution of the density distribution of the added component (nitrogen). The results show growing gas concentrations in the upper part of the bubble, extending from the bubble interface to the neck region. Along with the separated bubble, the accumulated gas is eventually removed from the surface.

**Video M4** | **Diffuse-interface simulations of bubble dynamics on a biphilic surface in a single-component system.** The results show the shrinkage and ultimate collapse of the vapour bubble, with the continuous pinning of the three-phase contact line at the boundary between the hydrophobic and hydrophilic subregions. Without dissolved nitrogen, the bubble growth is severely hindered by the strong condensation and cannot reach the minimum size threshold required for bubble pinch-off.
